# Ally or traitor: the dual role of p62 in caspase-2 regulation

**DOI:** 10.1038/s41419-024-07230-3

**Published:** 2024-11-14

**Authors:** Pavel I. Volik, Alexey V. Zamaraev, Aleksandra Y. Egorshina, Nikolay V. Pervushin, Anastasia A. Kapusta, Pyotr A. Tyurin-Kuzmin, Anastasia V. Lipatova, Thilo Kaehne, Inna N. Lavrik, Boris Zhivotovsky, Gelina S. Kopeina

**Affiliations:** 1 Engelhardt Institute of Molecular Biology, RAS, Moscow, Russia; 2 Faculty of Medicine, MV Lomonosov Moscow State University, Moscow, Russia; 3 Translational Inflammation Research, Medical Faculty, Center of Dynamic Systems (CDS), Otto von Guericke University, Magdeburg, Germany; 4 Division of Toxicology, Institute of Environmental Medicine, Karolinska Institutet, Stockholm, Sweden

**Keywords:** Cell biology, Biochemistry

## Abstract

Caspase-2 is a unique and conserved cysteine protease that is involved in several cellular processes, including different forms of cell death, maintenance of genomic stability, and the response to reactive oxygen species. Despite advances in caspase-2 research in recent years, the mechanisms underlying its activation remain largely unclear. Although caspase-2 is activated in the PIDDosome complex, its processing could occur even in the absence of PIDD1 and/or RAIDD, suggesting the existence of an alternative platform for caspase-2 activation. Here, we show that caspase-2 undergoes ubiquitination and interacts with scaffolding protein p62/sequestosome-1 (SQSTM1) under normal conditions and in response to DNA damage. p62 promotes proteasomal but not autophagic caspase-2 degradation as well as its dimerization and activation that triggers the caspase cascade and, subsequently, cell death. Inhibition of p62 expression attenuates cisplatin-induced caspase-2 processing and apoptosis. Notably, the ZZ domain of p62 is critical for caspase-2 binding, whereas the UBA domain is seemingly required to stabilize the p62–caspase-2 complex. Thus, we have uncovered the dual role of p62 in regulating caspase-2 activity: it can foster the degradation of caspase-2 in the proteasome or facilitate its activation by acting as a scaffold platform.

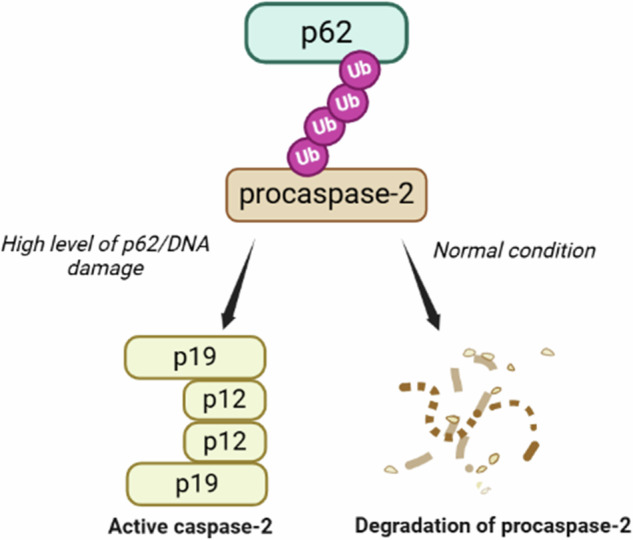

## Introduction

A family of cysteine proteases known as caspases is involved in multiple cellular processes chief among which is apoptosis, the most well-known type of programmed cell death (PCD) [1, 2]. Caspases are present in cells as inactive zymogens (procaspases), comprising an N-terminal prodomain and large (p20) and small (p10) catalytic subunits [3]. In response to extrinsic or intrinsic stimuli, initiator procaspases (procaspase-2,-8,-9,-10) are recruited to high-molecular-weight activation platforms that lead to proximity-induced dimerization and proteolytic autocleavage of procaspases, resulting in their activation [4].

Caspase-2 is the most evolutionarily conserved member of the caspase family. It has been implicated in both apoptotic and non-apoptotic signaling pathways, including tumor suppression, cell cycle regulation, genomic stability, and DNA repair [5–8]. Moreover, caspase-2 negatively regulates necroptosis and ferroptosis [9, 10]. A canonical procaspase-2 activation platform, the PIDDosome, is formed via interactions between the death domain of p53-induced protein with a death domain (PIDD1) and the death domain of the adapter protein receptor-interacting protein-associated ICH-1/CED-3 homologous protein with a death domain (RAIDD), whose N-terminal domain caspase activation recruitment domain (CARD) binds to the CARD domain of procaspase-2 [11–13]. The PIDDosome was initially described as a caspase-2 activation complex whose assembly occurs in response to DNA damage-inducing apoptotic cascade [11]. Later, researchers demonstrated that the PIDDosome could also assemble in the nucleolus upon genotoxic stress [14]. The PIDDosome also plays a role in processes not directly related to cell death such as regulation of the cell cycle and differentiation [15, 16].

Caspase-2 is activated regardless of PIDDosome formation in various experimental conditions [17]. It has been established that RAIDD, but not PIDD1, is essential for caspase-2-mediated neuronal apoptosis induced by neuronal growth factor (NGF) deprivation and amyloid beta (Aβ) treatment [18]. Consistent with these findings, DNA damage–induced processing of caspase-2 was not suppressed in primary lymphocytes from PIDD1-deficient mice. Moreover, active caspase-2 was detected in multiprotein complexes in extracts derived from PIDD1- or RAIDD-deficient SV40-immortalized mouse embryonic fibroblasts (MEFs) [19]. Additionally, caspase-2 cleavage was not prevented by depletion of either PIDD1 and RAIDD in response to α-toxin treatment [20]. Kopeina et al. [21] isolated a macromolecular protein complex containing catalytically active caspase-2 but not RAIDD from human ovarian cancer cells treated with the chemotherapeutic agent cisplatin. These findings indicate the existence of an alternative caspase-2 activating platform that might form upon DNA damage.

The interplay between cell death and cell survival signaling is crucial for cellular processes in normal and pathological conditions. If apoptosis is a ‘warrior’ of PCD induced by intracellular damage or external stimuli, then autophagy is a survival ‘scavenger’ that eliminates damaged components of cells. The well-known autophagic cargo adapter p62/sequestosome-1 (SQSTM1) is critical for crosstalk among these processes: p62 is tightly involved in the degradation of pro- and antiapoptotic proteins by autophagic and ubiquitin-proteasome systems [22]. Moreover, this protein can promote endoplasmic reticulum stress-induced apoptosis, acting as a scaffold for aggregation of ubiquitinated caspase-8 that induces its activation [23]. Researchers have also revealed that self-polymerization of p62 is required for the recruitment of ubiquitinated caspase-8 into aggresome-like structures and subsequent apoptosis induction upon ionizing radiation [24]. Given that caspase-2 could undergo tumor necrosis factor receptor-associated factor 2 (TRAF2)-mediated ubiquitination – a phenomenon that regulates its activity in the presence of intracellular stressors [25]—it can be assumed that ubiquitinated caspase-2 might potentially interact with p62 in a caspase-8 manner.

Here, we aimed to determine the possible role of p62 as an alternative caspase-2 activating platform or the main mediator of its degradation. First, we established that caspase-2 can be ubiquitinated in response to proteasome inhibition and treatment with cisplatin, a DNA-damaging agent. Using pull-down assays and mass spectrometry (MS), we revealed that caspase-2 interacts with p62 under physiological conditions and after DNA damage induction. Caspase-2 undergoes p62-dependent proteasomal degradation and can be activated in a p62-dependent manner upon cisplatin treatment. Furthermore, bimolecular fluorescence complementation (BiFC) demonstrated that full-length p62 promotes caspase-2 dimerization that leads to its activation. Pull-down assays and BiFC analysis revealed that the ZZ domain of p62 is critical for caspase-2 binding and dimerization, whereas the ubiquitin-associated (UBA) domain is involved in the stabilization of the p62–caspase-2 complex. Taken together, we have demonstrated for the first time the role of p62 in the control of caspase-2 activation and apoptosis induction.

## Results

### Ubiquitination of caspase-2 and its interaction with p62

**Fig. 1:**
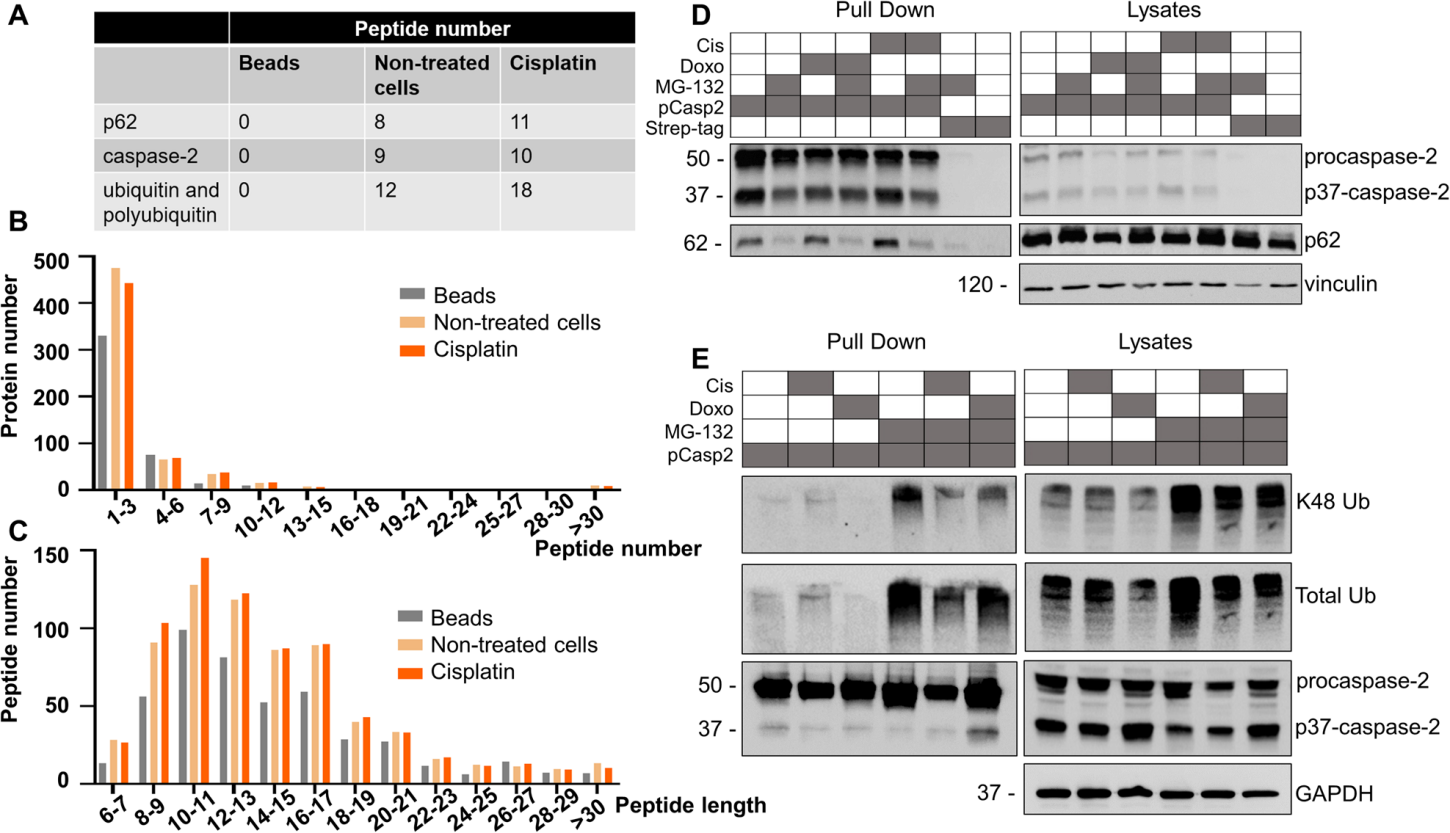
Ubiquitination of caspase-2 and its interaction with p62. **A** The proteins that co-precipitated with caspase-2 in normal conditions and after DNA damage induction and the number of identified peptides. **B**, **C** Quality control validation of mass spectrometry data: the **B** peptide number and **C** peptide length distributions. The averages of the three replicates are shown in histograms. **D** Strep-tag pull-down assay performed after overexpression of caspase-2-Strep-tag and subsequent treatment of HEK293T cells with 25 µM cisplatin or 2 µM doxorubicin for 18 h and/or 1 µM MG-132 for 6 h. The bands on the western blot corresponding to a cleaved fragment of caspase-2 are marked as p37-caspase-2. The pull-down with Strep-tag only was used as a control. **E** Ubiquitination of caspase-2 purified by Strep-tag pull-down assay. HEK293T cells were treated with 25 µM cisplatin or 2 µM doxorubicin for 18 h and/or 1 µM MG-132 for 6 h. When chemotherapeutic agents were combined with MG-132, the latter was added for 6 h before an 18 h incubation. One representative experiment from three independent experiments is shown. GAPDH glyceraldehyde 3-phosphate dehydrogenase. The gray box represents the presence of a treatment while the white box indicates its absence.

To identify potential interaction partners of caspase-2, we performed immunoprecipitation of caspase-2 from lysates of human embryonic kidney 293T (HEK293T) cells treated or not treated with 25 µM cisplatin for 18 h using an anti-caspase-2 antibody. MS analysis of the obtained samples identified several caspase-2-co-precipitated proteins, including p62 and components of the ubiquitination pathway such as ubiquitin and polyubiquitin, in lysates of HEK293T cells that had and had not been treated with cisplatin (Fig. 1A). Furthermore, we identified several caspase-2 substrates in the immunoprecipitations after cisplatin stimulation, confirming the specificity of the pull-down assay (Fig. S1). Quality control validation of the MS data is presented in Fig. 1B, C. We confirmed the interaction between caspase-2 and p62 with a Strep-tag pull-down assay, in which caspase-2 was coupled to Strep-tag (caspase-2-Strep-tag) (Fig. 1D). In these experiments, we also treated cells with cisplatin and doxorubicin for 18 h. Similarly to the MS analysis, there was an association between p62 and caspase-2 in these experiments. Caspase-2-Strep-tag overexpression led to its autocatalytic processing and generation of the catalytically active p37 fragment. It is worth noting that the p62 protein level did not change with caspase-2 overexpression. Treatment of cells with the proteasome inhibitor MG-132 increased the accumulation of p62 due to its engagement in proteasome degradation. There was a remarkable decrease in the level of p62 co-precipitated with caspase-2 in samples treated with MG-132. Inhibition of the proteasomal degradation system led to a total increase in ubiquitinated substrates. Of note, p62 normally binds to ubiquitin-marked targets, which results in the reduction of total p62 flux and the lack of free p62 molecules that are able to interact with caspase-2.

Given that we detected ubiquitin and polyubiquitin as potential binding partners of caspase-2 and considering that p62 commonly binds to ubiquitinated proteins, we elucidated whether caspase-2 undergoes ubiquitination under the above-mentioned conditions and performed a pull-down assay using caspase-2-Strep-tag (Fig. 1E). Treatment with cisplatin but not doxorubicin resulted in low caspase-2 ubiquitination. MG-132 promoted massive ubiquitination of caspase-2, including K48-linked ubiquitination, suggesting the possibility of proteasome-mediated degradation of this protease. To correctly estimate the levels of caspase-2 ubiquitination in pull-down probes, the normalization to procaspase-2 levels should be considered. Densitometric analysis of western blot shows no significant reduction in caspase-2 ubiquitination following DNA damage (Fig. S2). Taken together, caspase-2 interacts with p62 and can be ubiquitinated in response to cisplatin and MG-132 treatment.

### Caspase-2 undergoes proteasomal but not autophagic degradation

**Fig. 2:**
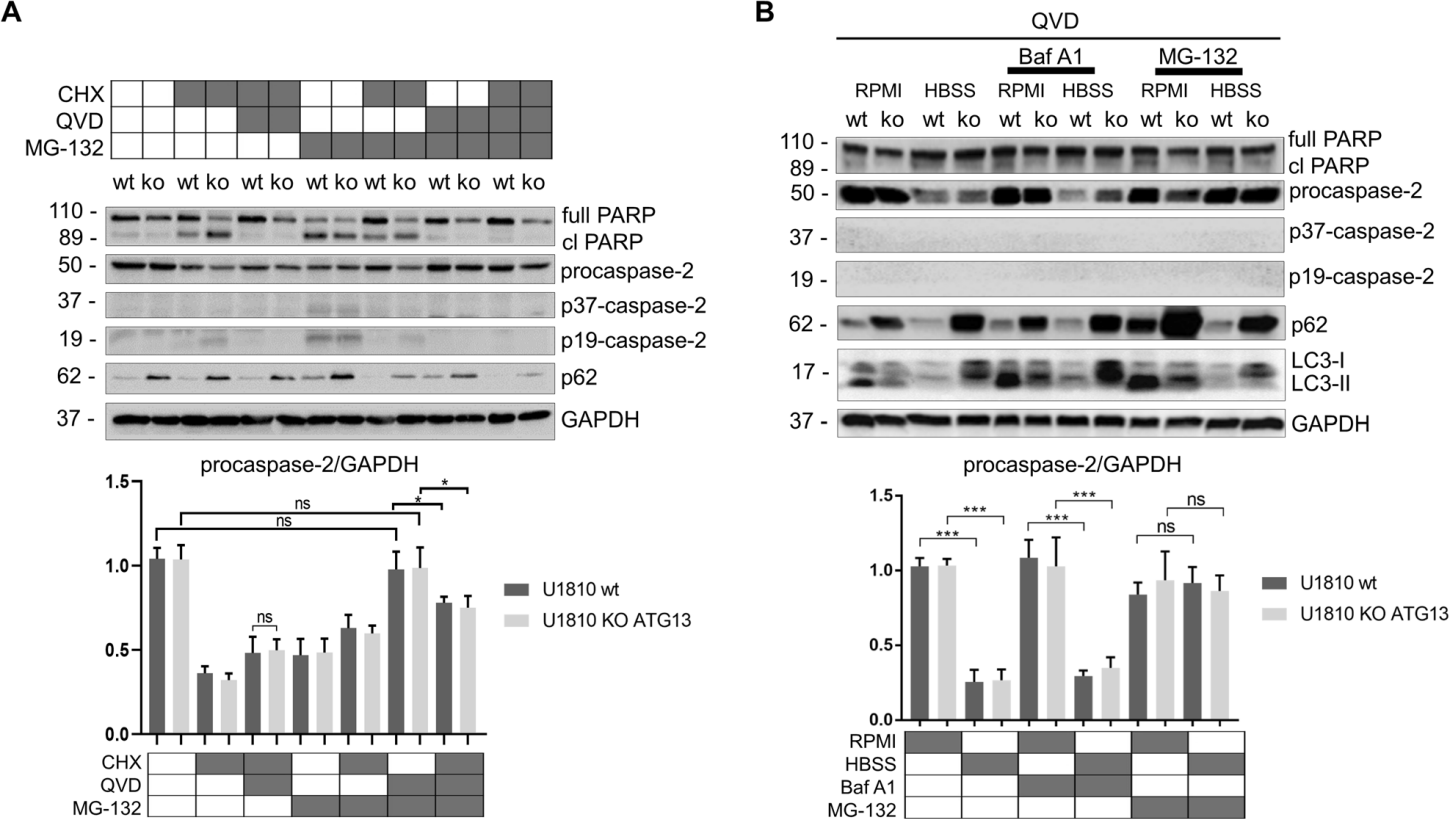
Caspase-2 undergoes proteasomal but not autophagic degradation. **A** Western blotting and densitometry of procaspase-2 levels in wild type (wt) and knockout (koATG13) U1810 cell lysates. Cells were treated with 5 µg/ml cycloheximide (CHX) for 18 h, 20 µM QVD for 24 h, and 1 µM MG-132 for 6 h. The bands on the western blot corresponding to cleaved fragments of caspase-2 are marked as p37-caspase-2 and p19-caspase-2. Cell death was estimated based on an accumulation of cleaved poly (ADP-ribose) polymerase (PARP, denoted as cl PARP). **B** Analysis of nutrient limitation–induced procaspase-2 degradation in wt and koATG13 U1810 cell lysates in the presence of QVD. Roswell Park Memorial Institute 1640 (RPMI1640) cell culture medium was used as a control. After growing in RPMI1640 or Hank’s balanced salt solution (HBSS), cells were treated with 25 nM bafilomycin A1 (BafA1) for 18 h or 1 µM MG-132 for 6 h. The observed conversion of LC3-I to LC3-II increased upon BafA1 treatment, confirming that this drug blocks basal autophagic flux. Caspase activation and apoptosis induction were suppressed with 25 µM QVD (added in all probes). Results from one out of three independent experiments are shown. The data are presented as the mean ± standard error of the mean of at least three independent experiments. Statistical analysis was performed using Student’s *t* tests; *p < 0.05. GAPDH glyceraldehyde 3-phosphate dehydrogenase. The gray box represents the presence of a treatment while the white box indicates its absence.

Because p62 is tightly involved in the autophagic and proteasomal degradation systems, it is important to determine which machinery is responsible for the degradation of ubiquitinated caspase-2. To elucidate the impact of autophagic degradation, we used the non-small cell lung cancer (NSCLC) line U1810 (wt) and U1810 cells with a deficiency of *ATG13* (koATG13), which are incapable of autophagy. Increased p62 indicated the suppression of autophagy in U1810 koATG13 cells (Fig. 2A). To inhibit proteasomal degradation and/or protein synthesis, we treated cells with MG-132, cycloheximide (CHX), or their combination. To exclude possible MG-132- and CHX-induced apoptosis [26, 27], which we evaluated based on the accumulation of cleaved poly (ADP-ribose) polymerase (PARP), we also treated cells with the pan-caspase inhibitor QVD. Western blotting clearly demonstrated that inhibition of translation by CHX in the presence of QVD led to a reduction in procaspase-2 protein levels in both wt and koATG13 U1810 cells, without substantial differences between the groups. Thus, caspase-2 mainly undergoes autophagy-independent degradation. In contrast, inhibition of the proteasomal degradation system by MG-132 in the presence of QVD resulted in the recovery of the procaspase-2 level, which was comparable to the procaspase-2 level in the non-treated cells (Fig. 2A). Treatment with MG-132, CHX, and QVD caused certain mitigation in the level of procaspase-2 due to protein synthesis interruption. The findings point to the key role of the proteasome in the regulation of the caspase-2 level in cells.

Next, the procaspase-2 level was analyzed after activating autophagy by nutrient limitation (Fig. 2B). For autophagy induction, wt and koATG13 U1810 cells were grown in amino acid and growth factor-free Hank’s balanced salt solution (HBSS) [28]. To rule out MG-132- and starvation-mediated apoptosis [29] accompanied by caspase-2 processing and the formation of the active cleaved p37 and p19 fragments of caspase-2, all cells were incubated with QVD. There was a decrease in the LC3I/II and p62 levels in the cells incubated in HBSS, revealing the induction of autophagy. We observed massive degradation of procaspase-2 without accumulation of the active p37 or p19 forms under nutrient restriction. This model also showed no difference in the procaspase-2 levels between wt and koATG13 U1810 cells. Under nutrient limitation, treatment with MG-132 and QVD facilitated full recovery of procaspase-2; however, treatment with the autophagy inhibitor bafilomycin A1 (BafA1) did not restore the procaspase-2 level. Analysis of procaspase-2 degradation under nutrient limitation in HEK293T cells treated with MG-132 or BafA1 showed similar results (Fig. S3). These data confirm that proteasomal but not autophagic machinery is involved in the degradation of caspase-2.

### p62 contributes to the proteasomal degradation of caspase-2 as well as its activation and apoptosis induction

**Fig. 3:**
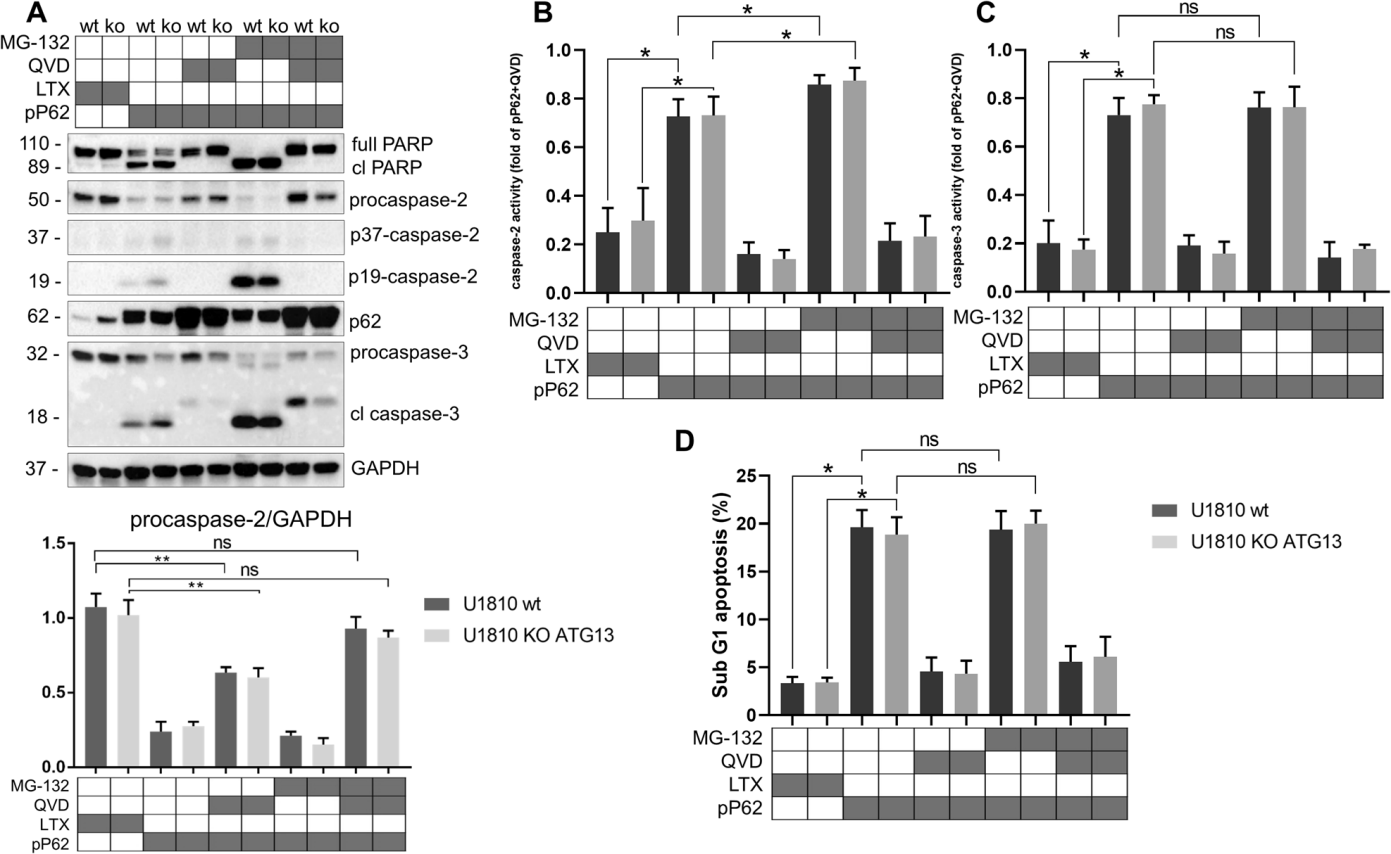
p62-dependent caspase-2 proteasomal degradation and activation. **A** Analysis of procaspase-2 levels by western blotting and densitometry in wild type (wt) and knockout (koATG13) U1810 cells transfected with genetic construction encoding *SQSTM* (pP62). Caspase activation was suppressed by 20 µM QVD. MG-132 (1 µM) was used for proteasomal inhibition (6-h incubation time). Apoptosis was evaluated based on the accumulation of cleaved caspase-3 and poly (ADP-ribose) polymerase (PARP). Overexpression of p62 enhanced **B** caspase-2 and **C** caspase-3 peptidase activities, as measured using substrates Ac-VDVAD-AMC and Ac-DEVD-AFC, respectively, in lysates of wt and koATG13 U1810 cells with overproduction of p62. Both cell lines were treated with 1 µM MG-132 for 6 h and/or 20 µM QVD for 24 h, and then caspase activity was measured. The histograms present the slope of the enzymatic activity curve. **D** Assessment of the SubG1 population of wt and koATG13 U1810 cells overexpressing p62. The data are presented as the mean ± standard error of the mean of at least three independent experiments. Statistical analysis was performed using Student’s *t*-test; *p < 0.05. GAPDH glyceraldehyde 3-phosphate dehydrogenase. The gray box represents the presence of a treatment while the white box indicates its absence.

We investigated the role of p62 in the proteasomal degradation of caspase-2 by transfecting wt and koATG13 U1810 cells with a plasmid encoding the *SQSTM1* gene. p62 overexpression stimulated activation of caspase-2 accompanied by the formation of its catalytically active fragments and apoptosis induction, indicated by cleavage of the apoptotic markers, caspase-3 and PARP (Fig. 3A). QVD inhibited the p62-dependent processing of caspase-2, but the level of procaspase-2 did not recover completely. Thus, procaspase-2 degradation involves the proteasomal system. Consistently, the procaspase-2 protein level was fully restored when the proteasomal system was inhibited by MG-132. It is important to note that p62 overexpression influences the procaspase-2 protein level and does not affect caspase-2 gene transcription, as evidenced by the real-time polymerase chain reaction (qPCR) data (Fig. S4). Thus, increased p62 level promoted proteasomal degradation of caspase-2 as well as its activation, resulting in enhanced activity of caspase-2 as well as caspase-3 and increased cell death (Fig. 3B–D). p62 overexpression in HEK293T cells had the same effects (Fig. S5).

### Downregulation of p62 leads to decreased caspase-2 activation and attenuates the caspase cascade upon cisplatin treatment

**Fig. 4:**
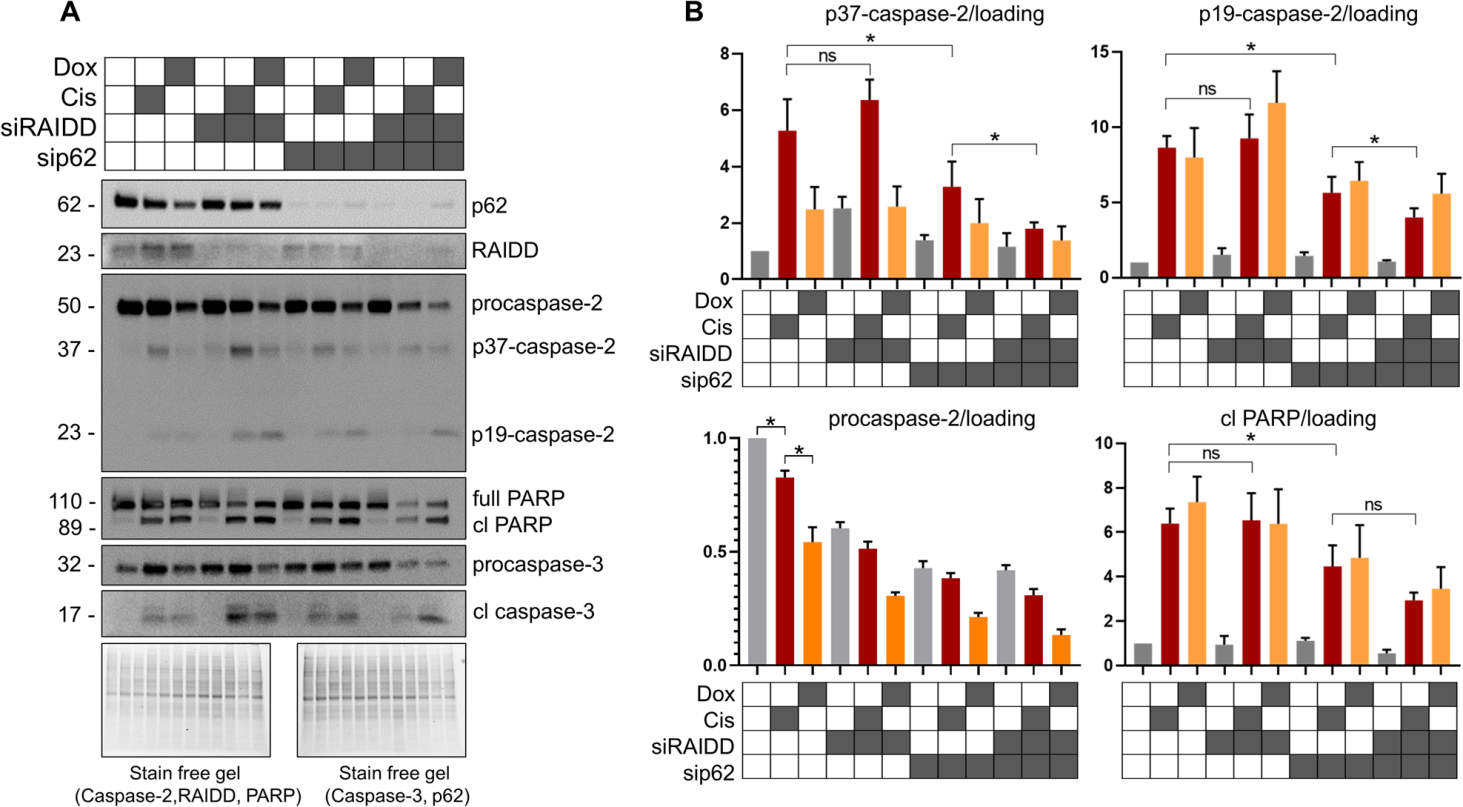
p62 promotes caspase-2 processing and apoptosis upon DNA damage. **A** U1810 cells were transfected with sip62/siRAIDD or non-targeting siRNA as a negative control. On the next day, cells were treated with 25 µM cisplatin or 2 µM doxorubicin for 18 h. Apoptosis was evaluated by the accumulation of cleaved forms of caspase-3 and poly (ADP-ribose) polymerase (PARP). **B** Densitometry of western blot bands corresponding to procaspase-2, cleaved active caspase-2 fragments (p37-caspase-2 and p19-caspase-2), and products of PARP cleavage (cl PARP). The data are presented as the mean ± standard error of the mean of at least three independent experiments. Statistical analysis was performed using Student’s *t*-test; *p < 0.05. Abbreviations: GAPDH, glyceraldehyde 3-phosphate dehydrogenase; RAIDD, receptor-interacting protein-associated ICH-1/CED-3 homologous protein with a death domain. The gray box represents the presence of a treatment while the white box indicates its absence.

Previous studies have suggested the existence of an alternative caspase-2 activation complex in response to DNA damage [21]. Therefore, we determined the role of p62 in caspase-2 processing and the caspase cascade induced by cisplatin and doxorubicin. Specifically, p62 expression was suppressed by transfecting cells with small interfering RNA (siRNA, namely sip62). RAIDD expression was also silenced using siRAIDD to interrupt PIDDosome assembly, which is reported to be a canonical caspase-2 activation complex upon DNA damage [11]. Western blotting showed the sufficient reduction of RAIDD and p62 levels by siRNA (Fig. 4A). Accumulation of processed caspase-3 and cleaved PARP in cells treated with DNA damage-inducing agents indicated the induction of apoptosis (Fig. 4A). Based on western blotting, p62 downregulation was associated with decreased accumulation of active caspase-2 fragments, as well as active caspase-3 and cleaved PARP upon cisplatin treatment (Fig. 4B). In contrast, p62 had a trivial impact on caspase-2 activation in response to doxorubicin treatment. Moreover, in contrast to cisplatin, doxorubicin stimulated a fast and marked conversion of procaspase-2 into the active p19 fragment, suggesting that caspase-2 activation may occur differently depending on the DNA-damaging agent. Interestingly, RAIDD downregulation did not affect caspase-2, caspase-3, and PARP cleavage upon DNA damage. This outcome could be explained by the negligible role of the PIDDosome as a caspase-2 activation platform under these experimental conditions. Nevertheless, suppression of both p62 and RAIDD hampered caspase-2 processing more than only p62 inhibition, indicating the existence of some cooperation between the PIDDosome and p62 (Fig. 4). These data collectively revealed that p62 can stimulate caspase-2 activation and cell death in the context of cisplatin-induced DNA damage.

### p62 facilitates caspase-2 dimerization

**Fig. 5:**
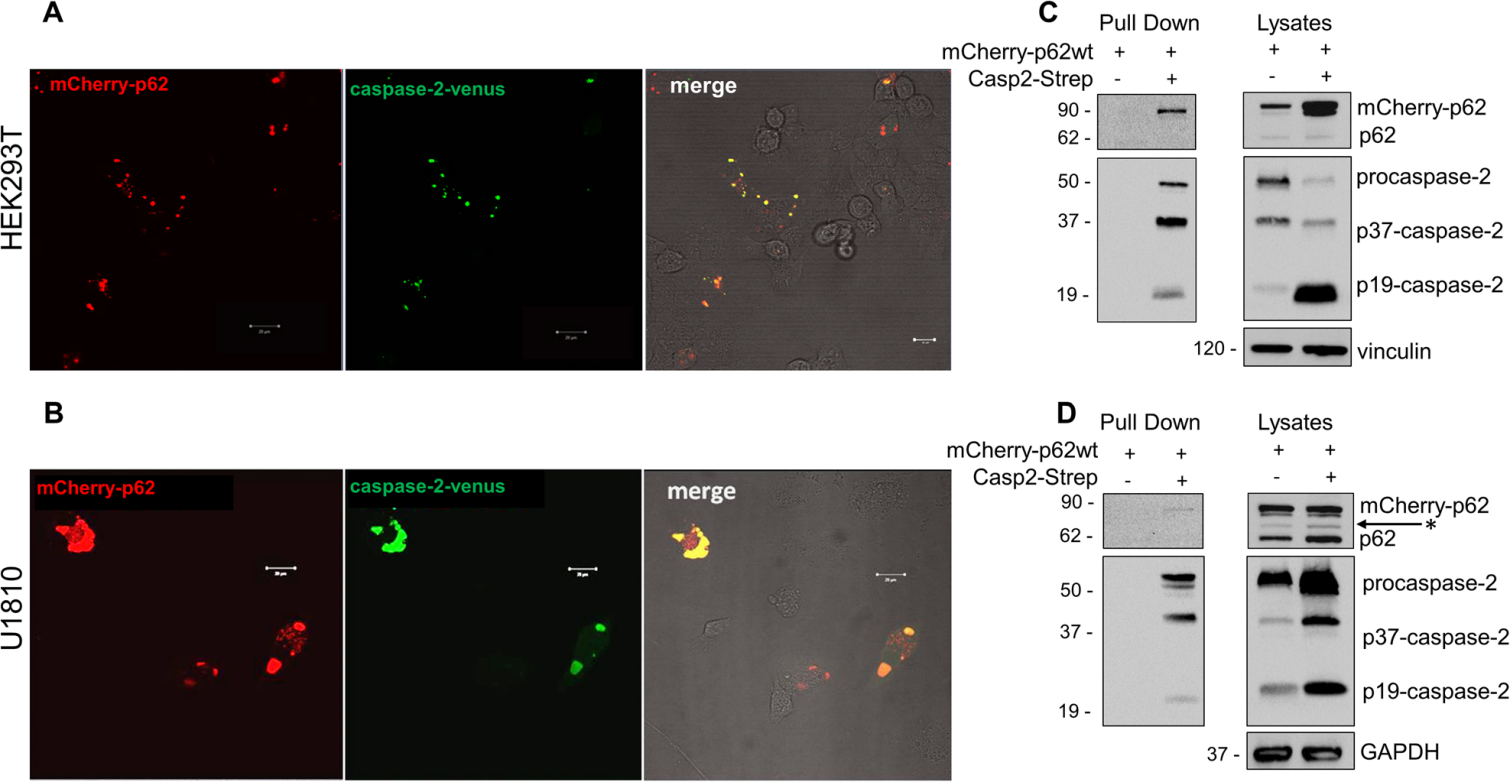
Caspase-2 dimerization is induced by p62. **A** HEK293T and **B** U1810 cells were transiently transfected with Casp2-CARD VN and Casp2-CARD VC along with the plasmid encoding mCherry-p62. Representative confocal micrographs of cells at 24 h after transfection are shown. Venus-positive foci are *green*, while mCherry-p62-positive foci are *red*. The scale bars represent 20 μm. Pull-down assay of lysates of **C** HEK293 T and **D** U1810 cells transfected with mCherry-p62 and/or Caspase-2-strep-tag (Casp2-strep). The bands on a western blot corresponding to a cleaved fragment of caspase-2 are marked as p37-caspase-2 and p19-caspase-2. * indicates a non-specific band.

It is known that caspase-2 dimerization is essential for its processing. Hence, to elucidate the role of p62 in caspase-2 activation, caspase-2 proximity was measured in real-time in living cells with p62 overexpression using a BiFC assay. Non-fluorescent fragments of the fluorescent protein Venus (VN and VC) were fused and assembled to form the fluorescent complex to the caspase-2 prodomain containing CARD, which is responsible for caspase-2 dimerization [30]. To confirm the p62-mediated dimerization of caspase-2, U1810 and HEK293T cells were transfected with caspase-2-Venus and mCherry-p62. Fluorescent confocal microscopy showed that caspase-2-Venus formed a fluorescent complex that co-localized with mCherry-p62, indicating an interaction between caspase-2 and p62 (Fig. 5A, B). mCherry overexpression did not lead to caspase-2-Venus association, confirming that caspase-2 proximity is induced by p62 (Fig. S6). To exclude potential cleavage of caspase-2 by caspase-8, which can undergo p62-mediated activation [23, 24, 31], we used SK-N-BE cells, a neuroblastoma cell line characterized by loss of caspase-8 expression [32]. After transfection of SK-N-BE cells with mCherry-p62 and caspase-2-Venus, we observed caspase-2-Venus fluorescence, verifying caspase-8-independent caspase-2 dimerization (Fig. S7). A caspase-2-Strep-tag pull-down assay proved that p62 and caspase-2 can be co-precipitated, providing additional evidence for an interaction between these proteins. Accumulation of catalytically active caspase-2 fragments highlighted the involvement of p62 in the processing of procaspase-2 (Fig. 5C, D). Thus, a high level of p62 is able to stimulate caspase-2 dimerization in living cells through interaction with this protease. Next, the impact of DNA-damaging agents and proteasome inhibition on the dimerization of caspase-2 was evaluated. The BiFC assay demonstrated that in response to doxorubicin, there was pronounced caspase-2 dimerization that did not depend on p62 overexpression and/or proteasomal inhibition by MG-132. In contrast, cisplatin treatment led to a negligible dimerization of caspase-2, which gradually increased with p62 overexpression and proteasomal inhibition (Fig. S8). According to the analysis of ubiquitination (Fig. 1C), doxorubicin did not promote the accumulation of ubiquitinated caspase-2. In the same conditions, p62 could not increase the dimerization of the caspase-2 prodomain. These findings suggest that there are ubiquitin-dependent mechanisms of caspase-2 activation via its interaction with p62.

### The PIDDosome is not involved in p62-mediated caspase-2-Venus dimerization

**Fig. 6:**
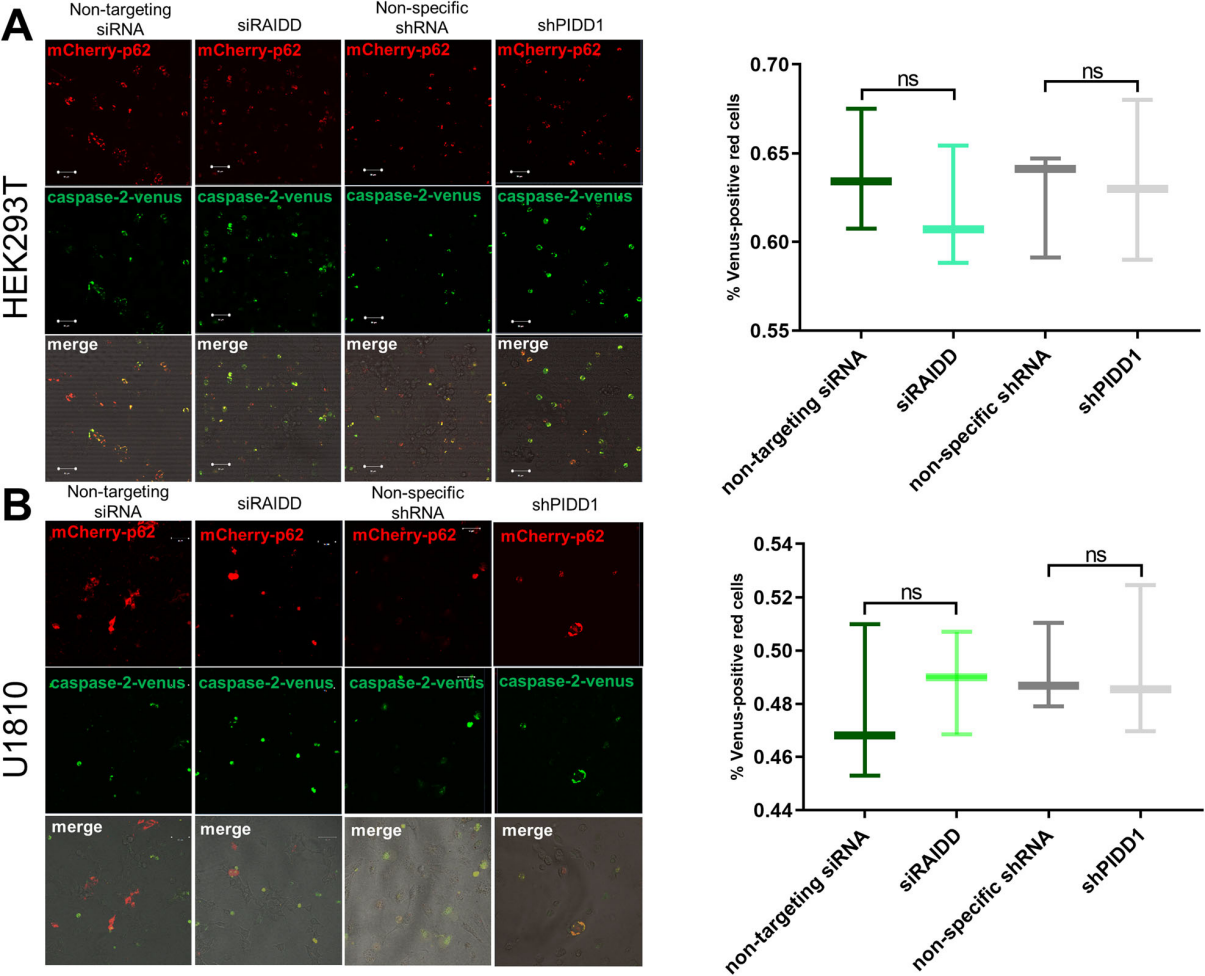
Dimerization of caspase-2 stimulated by p62 occurs regardless of the PIDDosome in. **A** HEK293T and **B** U1810 cells. To suppress receptor-interacting protein-associated ICH-1/CED-3 homologous protein with a death domain (RAIDD) expression, cells were transfected with siRAIDD. Non-targeting siRNA was used as a negative control. Cells that stably expressed shPIDD1 were used to exclude a p53-induced protein with a death domain 1 (PIDD1) action. Cells expressing non-specific shRNA were used as a negative control. Casp2-CARD VN and Casp2-CARD VC along with the plasmid encoding mCherry-p62 were added. Representative confocal micrographs of cells at 24 h after plasmid transfection are shown. The scale bars represent 50 μm. The percentage of mCherry-p62-positive (*red*) cells that were Venus-positive (*green*) was determined. At least 50 cells were analyzed, and the experiment was performed in triplicate. The results represent triplicate counts, with the error bars representing the standard deviation.

We aimed to establish whether dimerization and activation of caspase-2 induced by p62 depend on the formation of the PIDDosome, a canonical caspase-2 activation platform. For this purpose, the expression of RAIDD was suppressed by using siRAIDD and PIDD1 - by using shPIDD1 (Fig. 6) in HEK293T and U1810 cells before transfection with caspase-2-Venus and mCherry-p62. Western blotting indicated that siRAIDD and shPIDD1 almost completely suppressed RAIDD and PIDD1 expression, respectively (Fig. S9). It is worth noting that inhibition of PIDD1 seemingly induced a dramatic increase in PIDD isoform 2 (PIDD2) in HEK293T cells, which has the same prosurvival function as PIDD1 but is unable to act as a scaffold for PIDDosome formation [33]. BiFC analysis demonstrated that RAIDD or PIDD1 inhibition did not affect caspase-2 dimerization modulated by p62 overexpression in both cell lines. Thus, p62 can act as an alternative platform for caspase-2 activation, and this platform functions regardless of whether the PIDDosome has formed.

### The ZZ domain of p62 is crucial for interaction with caspase-2

**Fig. 7:**
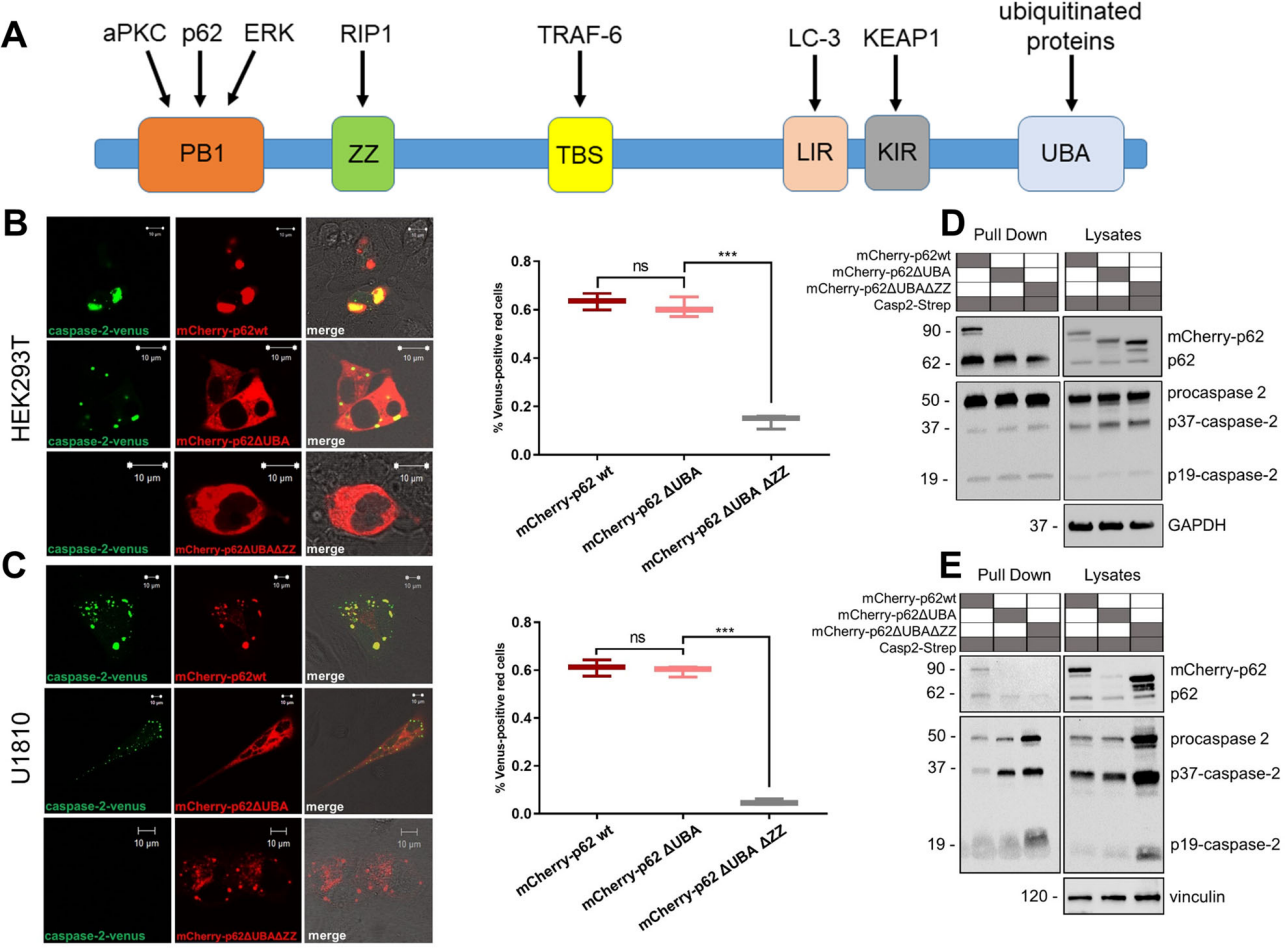
The ZZ domain of p62 is essential for p62–caspase-2 complex formation. **A** The domain structure of p62 and its interacting partners. **B** HEK293T and **C** U1810 cells were transiently transfected with Casp2-CARD VN and Casp2-CARD VC along with the plasmids encoding mCherry-p62, mCherry-p62ΔUBA, or mCherry-p62ΔUBAΔZZ. Representative images of cells 24 h after transfection are shown. The scale bars represent 10 μm. The percentage of mCherry-p62-positive (*red*) cells that were Venus-positive (*green*) was determined. At least 50 cells were analyzed, and the experiment was performed in triplicate. The results represent triplicate counts, with the error bars representing the standard deviation. Caspase-2-Strep-tag pull-down assay of lysates of **D** HEK293T and **E** U1810 cells transfected with Casp2-Strep and mCherry-p62, mCherry-p62ΔUBA, or mCherry-p62ΔUBAΔZZ. The bands on the western blot corresponding to cleaved fragments of caspase-2 are marked as p37-caspase-2 and p19-caspase-2.

Given that p62 could be involved in the proteasomal degradation of ubiquitinated caspase-2, we hypothesized that p62 binds to this protease through ubiquitin. p62 could act as an adapter protein that modulates protein-protein interactions via its protein-protein interaction domains (Fig. 7A). The PB1 domain is responsible for p62 self-oligomerization and is also involved in the binding of p62 to atypical protein kinase C (αPKC) or extracellular signal-regulated kinase 1 (ERK1). The zinc finger (ZZ) and TRAF6-binding (TBS) domains interact with RIP1 and TRAF6, respectively, to modulate NF-κB signaling. The C-terminal LC3-LC3-interacting region (LIR) and UBA of p62 play a key role in the selective autophagic degradation of ubiquitinated substrates. The Keap-interacting region (KIR) can interact with KEAP1, leading to the nuclear translocation of NRF2, a transcription factor implicated in antioxidant response [34]. Two of these domains, ZZ and UBA, display ubiquitin‐binding activity [35]. To investigate the role of the ZZ and UBA domains of p62 in caspase-2 binding, we generated constructs encoding mCherry fused to p62 with depletion of the UBA domain or the ZZ and UBA domains: mCherry-p62ΔUBA and mCherry-p62ΔUBAΔZZ, respectively.

BiFC analysis demonstrated that mCherry-p62ΔUBAΔZZ but not mCherry-p62ΔUBA was unable to promote caspase-2 dimerization (Fig. 7B, C). Interestingly, depletion of the UBA domain led to the smearing of mutant p62 localization all over the cell in contrast to full-length p62, which accumulated in distinct foci. However, a pull-down assay showed that both recombinant proteins, mCherry-p62ΔUBA and mCherry-p62ΔUBAΔZZ, could not be detected in caspase-2 samples, unlike mCherry-p62wt (Fig. 7D, E). Densitometry for western blotting with U1810 samples is shown in Fig. S10. Taken together, the ZZ domain is crucial for the interaction between p62 and caspase-2, whereas the UBA domain is seemingly involved in the stabilization of the p62–caspase-2 complex.

## Discussion

In this study, we examined p62 as a potential novel caspase-2 activation platform and investigated the mechanism underlying the interaction between p62 and caspase-2. To this end, we first established a key role for p62 in caspase-2 activation and degradation. It seems that p62 is able to bind to unwanted procaspase-2, which undergoes K48-linked ubiquitination and shuttling to the proteasome. Upon DNA damage, p62 could potentially oligomerize through the PB1 domain to foster proximity, dimerization, and activation of attached ubiquitinated procaspase-2. Given that oxidative stress promotes p62 self-oligomerization [36], it is quite possible that the p62 association can be also caused by cisplatin-induced oxidative stress [37]. Upon doxorubicin treatment, caspase-2 activation almost did not depend on p62 (Fig. 4). Thus, under doxorubicin treatment, the p62–caspase-2 complex is not formed, as confirmed by the absence of caspase-2 ubiquitination (Fig. 1). Caspase-2 ubiquitination is crucial for the interaction with p62 because deletion of the UBA and ZZ domains, which are responsible for interacting with ubiquitinated proteins, completely destroys the ability of p62 to bind caspase-2 (Fig. 7). We confirmed the assumption that caspase-2 could be activated in different ways upon doxorubicin and cisplatin treatment based on the faster and more robust conversion of procaspase-2 into an active p19 fragment in the presence of doxorubicin (Fig. 4), as well as a higher degree of dimerization of caspase-2 in cells treated with doxorubicin compared with cells treated with cisplatin (Fig. S8). This is quite logical because these DNA-damaging agents have different mechanisms of action. We also showed that p62 upregulation can stimulate caspase-2 activation. It is important to note that caspase-2 processing occurs only when p62 expression is drastically enhanced, as evidenced by the absence of accumulation of active caspase-2 fragments in koATG13 U1810 cells in which p62 is moderately elevated (Fig. 3A).

**Fig. 8:**
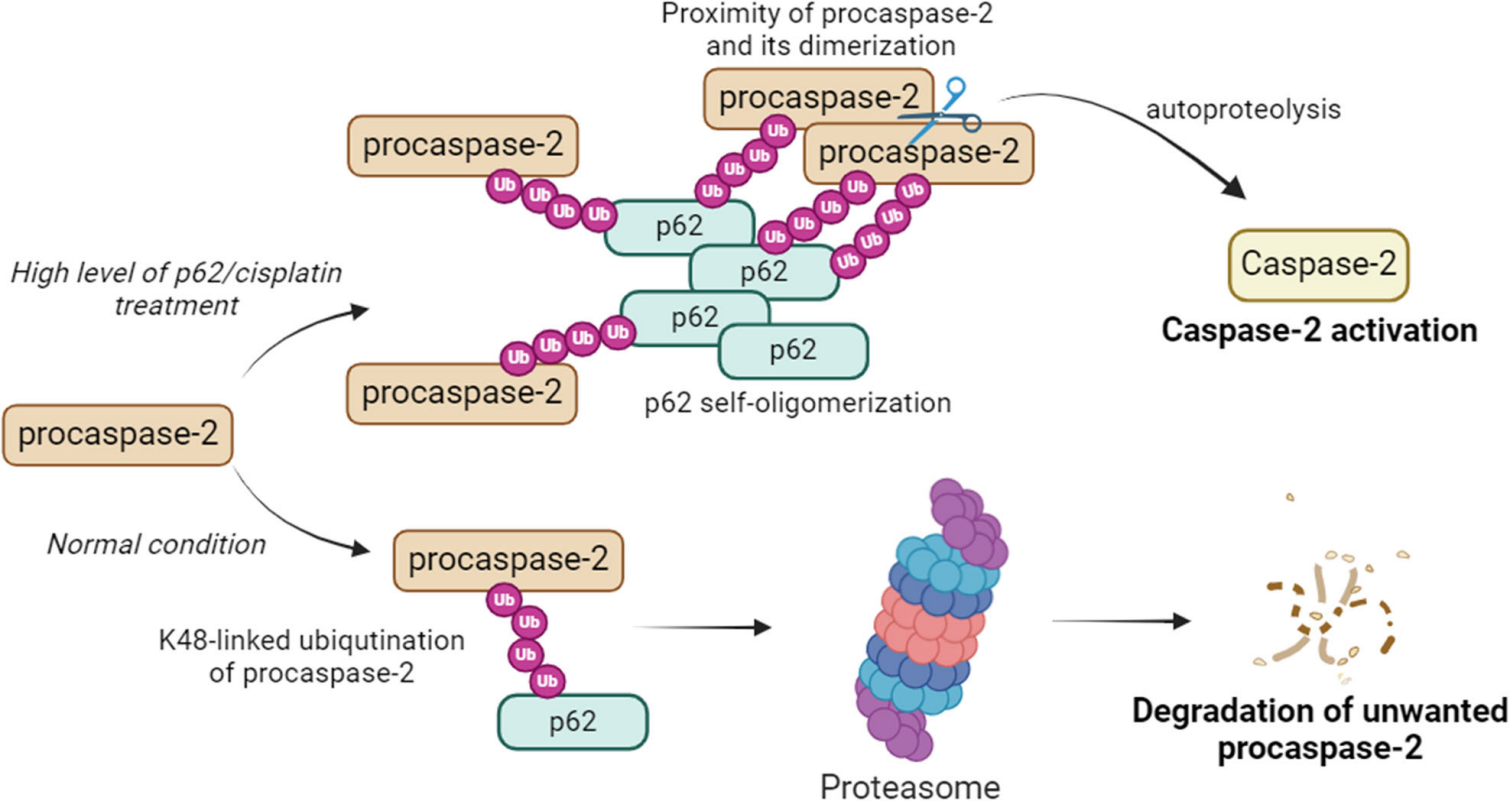
The putative mechanism of caspase-2 regulation by p62. K48-ubiquitinated procaspase-2 can interact with p62, which transports it to the proteasome to degrade unwanted enzymes. A high level of p62 and cisplatin treatment results in p62 oligomerization that promotes proximity and dimerization of ubiquitinated caspase-2, leading to its subsequent activation. The illustration was generated using BioRender.

Our pull-down assay and BiFC approach demonstrated that the ZZ domain of p62 is critical for caspase-2 binding, whereas the UBA domain is apparently responsible for stabilizing the p62–caspase-2 complex, probably because ubiquitin binding to the ZZ domain can sterically interfere with the PB1 domain‐mediated oligomerization. Remarkably, the UBA domain but not the ZZ domain plays a prominent role in the interaction between p62 and ubiquitinated caspase-8 [24]. Ubiquitin‐binding activity displayed by the ZZ and UBA domains of p62 as well as ubiquitination of caspase-2 upon cisplatin treatment suggest that p62 facilitates caspase-2 activation by binding to it via ubiquitin chains. The possible mode of caspase-2 regulation by p62 is shown in Fig. 8. However, how p62 switches between facilitating caspase-2 activation and its proteasomal degradation requires further research.

We also found that p62-mediated caspase-2 processing and apoptosis occur in a PIDDosome-independent manner, pointing to the putative engagement of p62 in caspase-2 activation and cell death stimulation in PIDD1-deficient cells, PIDD1- and RAIDD-knockout MEFs and PIDD1-null mice described in previous studies [17–20]. However, we observed a certain accumulation of active caspase-2 fragments upon DNA damage even when p62 and the PIDDosome had been inhibited. In this scenario, caspase-2 could be cleaved by other caspases (probably caspase-8) that are activated independently of caspase-2 after apoptosis induction. Thus, after DISC formation, caspase-8 is able to cleave and activate caspase-2 [38].

Another intriguing finding is the impact of p62 on caspase-2 in a non-cancerous cell line (HEK293T) as well as two tumor cell lines (U1810 and SK-N-BE), indicating the generality of this p62-dependent caspase-2 regulatory avenue. It is well known that caspase-2 acts as a tumor suppressor in many cancers, including lung cancer [39], whereas p62 overexpression and/or its reduced degradation is commonly associated with tumor formation, cancer promotion, and resistance to therapy [40]. Indeed, accumulation of p62 is related to a poor prognosis in patients with lung adenocarcinoma [41]. Such a pro-oncogenic function of p62 indicates a major role of p62-mediated caspase-2 proteasomal degradation in lung cancer that is confirmed by excess proteasome activity consistent with proteotoxic stress that has been observed in many tumor cell types expressing oncogenic mutant p53 alleles, including NSCLC [42]. These arguments emphasize the great potential of p62 inhibition strategies for cancer treatment. However, p62 might act as a tumor suppressor in esophageal adenocarcinoma and colorectal cancer under certain circumstances [43, 44], indicating that an increased level of p62, leading to caspase-2 activation, could improve the prognosis of several tumor types. In addition, high expression of p62 and caspase-2 correlates with poor survival outcomes in patients with liver hepatocellular carcinoma (Fig. S11). This is consistent with recent studies uncovering a key role of caspase-2 in lipotoxicity-induced apoptosis in hepatocytes, promoting the development of non-alcoholic steatohepatitis (NASH) that evolves into hepatocellular carcinoma [45–47]. Perturbation of p62 activity is also related to the pathogenesis of several liver diseases. p62 has been established as a crucial component of protein inclusions, which have frequently been detected in biopsy samples from patients with NASH and hepatocellular carcinoma [48]. Although the abundance of p62 aggregates seems to predict the development of these liver diseases, the mechanisms by which p62 contributes to the progression of these diseases remain unclear. There is reason to suppose that the effects we observed may be related to the p62-mediated activation of caspase-2 in hepatocytes.

Described p62-mediated caspase-2 modulation could be an intriguing option for the regulation of autophagy as both proteins are tightly involved in this process. If p62 is a classical receptor of autophagy [34], then caspase-2 might inhibit autophagy by preventing the accumulation of reactive oxygen species, whose overproduction causes the dissociation of the mammalian target of rapamycin complex (mTORC) from the autophagy-initiation complex [49]. Thus, activation of caspase-2 by p62 could potentially diminish tumorigenesis by reducing autophagy. Nevertheless, regulation of autophagy by caspase-2 remains a mostly unexplored area. For example, it is unclear whether caspase-2 could also inhibit autophagy in tumor cells and whether this biological effect of caspase-2 is critical for tumor suppression. It is important to understand these complex processes and crosslinks between autophagy and apoptosis because their interplay determines whether a cell will live or die which underlies the therapeutic outcomes and disease progression.

Thus, we are the first group to reveal p62 as a novel caspase-2 regulator: it could interact with caspase-2 to promote its proteasomal or activation, and thus increase cell death, in normal conditions or under genotoxic stress. We demonstrated that the ZZ domain of p62 plays a key role in p62–caspase-2 complex formation. We observed the regulatory mechanism in non-cancerous and tumor cell lines. Our findings provide new insights into regulatory networks of apoptotic cell death and could have potential significance for the prognosis of anti-cancer therapy efficacy.

## Materials and methods

### Cell culture

HEK293T cells (obtained from ATCC, USA) and human lung adenocarcinoma U1810 and neuroblastoma SK-N-BE cells (from the collection of Uppsala University, Sweden) were cultured in Dulbecco′s Modified Eagle′s Medium (DMEM, Gibco, USA) supplemented with 10% (w/v) heat-inactivated fetal bovine serum (Gibco), 100 μg/ml penicillin, and 100 μg/ml streptomycin (Gibco). For HBSS starvation, cells were incubated in HBSS (Gibco) for 3 h. For all experiments, cells were grown in a humidified 5% CO_2_ atmosphere at 37 °C and maintained in the logarithmic growth phase. Throughout the experiments, cells were treated with 25 µM cisplatin (Teva, Israel), 2 µM doxorubicin (Sigma-Aldrich, USA), 5 µg/ml CHX (Sigma-Aldrich), 20 µM QVD-OPH (MP Biomedicals, USA), 1 µM MG-132 (Sigma-Aldrich), and/or 25 nM bafilomycin A1 (Sigma-Aldrich).

### Cloning experiments

The pESG-IBA103 plasmid (IBA-Lifesciences, Germany) is used for highly productive gene expression in mammalian cells and contains a Strep-tag for efficient purification through affinity chromatography. Based on the pcDNA3-caspase-2 plasmid, a PCR fragment encoding the full-length caspase-2 sequence was obtained. It was isolated with the Gene JET Gel Extraction Kit (Fermentas, USA) and cloned according to the StarGate Direct Transfer Cloning kit (IBA Lifesciences) into the pESG-IBA103 plasmid as described earlier [50]. *Escherichia coli* XL-1 Blue cells were transformed using a ligase mixture according to a standard technique. The correct sequence of the obtained plasmid DNA was confirmed by sequencing.

The pDEST-mCherry-p62 plasmid, a gift from the Department of Neurology, Washington University School of Medicine, St. Louis, MO, USA [51], was used to overexpress full-length p62 fused to mCherry at the N-terminus. To generate the mCherry-p62ΔUBA mutant, a PCR fragment encoding mCherry-p62 without the UBA domain was obtained based on the pDEST-mCherry-p62 plasmid and cloned into the pVAX-1 expression vector. This pVAX-1-mCherry-p62ΔUBA plasmid was used to generate the pVAX-1-mCherry-p62ΔUBAΔZZ plasmid by deleting the sequence encoding the ZZ domain of p62. The correct sequence of the obtained plasmid DNA was confirmed by sequencing.

### Generation of cell lines with knockout and stable knockdown of the target genes

The koATG13 U1810 cell line was generated as described previously [52]. Lentiviral plasmids expressing shRNA to PIDD1 were obtained by sticky-end cloning (BamH1 and EcoR1) of double-stranded phosphorylated sites (annealed from the single-stranded sequences shown in Table 1) into the pLKO-puro vector. The shRNA nucleotide sequences are presented in Table 1. Then, the plasmids were used to produce lentiviral stocks as described previously [53]. Briefly, HEK293T cells were seeded into 6-well plates at 5 × 10^5^ cells per well and incubated at 37 °C with 5% CO_2_ for 24 h to achieve 70% confluence. The transfection reagent Lipofectamine 3000 (Thermo Scientific) was used to transfect the packaging and target plasmids; the lentiviral stocks were collected from 24 to 72 h. After filtering through a 0.45 μm filter, the effluent was cryopreserved and used as needed. The lentiviral titer was determined in HEK293T by using the final dilution approach. Subsequent selection on puromycin was carried out according to standard methods 3 days after transduction. For efficient cell transduction, the multiplicity of infection was 1–5.

**Table 1:** Small hairpin RNA (shRNA) nucleotide sequences.

#	Name	Sequense
1	PIDD1-1-dir	5′phospho-GATCGAGGAGATGCTTCAGAGGATTCACGTG AATCCTCTGAAGCATCTCCTTTTTTG
2	PIDD1-1-rev	5′phospho-AATTCAAAAAAGGAGATGCTTCAGAGGATTCACGTG AATCCTCTGAAGCATCTCCTCG
3	PIDD1-2-dir	5′phospho-GATCGAACCGGCTGAGCTTGGACCTCACGTG AGGTCCAAGCTCAGCCGGTTTTTTTG
4	PIDD1-2-rev	5′phospho-AATTCAAAAAAAACCGGCTGAGCTTGGACCTCACGTGA GGTCCAAGCTCAGCCGGTTCG
5	PIDD1-3-dir	5′phospho-GATCGGTGGAATTCTTGCGTCTGAGCACGTG CTCAGACGCAAGAATTCCACTTTTTG
6	PIDD1-3-rev	5′phospho-AATTCAAAAAAGTGGAATTCTTGCGTCTGAGCACGTGC TCAGACGCAAGAATTCCACCG

### Plasmid and siRNA transfection

Cells were transfected with the designated plasmids by using Lipofectamine LTX/Plus (Life Technologies, Germany), or with siRAIDD (sense chain: AAucuuGAcGGAAAAccAudTsdT; antisense chain: AUGGUUUUCCGUcAAGAUUdTsdT) and sip62 (sense chain: cuuccGAAucuAcAuuAAATsT; antisense chain: UUuAAUGuAGAUUCGGAAGTsT) by Lipofectamine RNAiMAX according to the manufacturer’s instructions. Six hours after transfection, the medium was changed, and drugs were administered.

### qPCR

RNA was isolated using the TRIzol reagent (Invitrogen, USA). One microgram of total RNA was reverse-transcribed into complementary DNA (cDNA) using the iScript cDNA Synthesis Kit (Bio-Rad, USA). qPCR was performed using gene-specific primers and SYBR Green Supermix (Bio-Rad) in a 20 μl volume in triplicate on a CFX96 Real-Time PCR machine (Bio-Rad). Analysis was performed using the CFX Manager software (Bio-Rad). The expression values were based on 10-fold serial dilutions of standards and normalized to the *RPL13A* level. The primers for qPCR are presented in Table 2.

**Table 2:** Primers for qPCR.

Gene	Primer (5′-3′)
Casp2	F: CACTGGTGTTGAGCAATGTG R: GCTGAAAAACCTCTTGGAGC
RPL13A	F: AAGGTCGTGCGTCTGAAGCCT R: ACGTTCTTCTCGGCCTGTTTCCGT

### Flow cytometry

Twenty-four hours after transfection with a plasmid encoding *SQSTM1* in the presence or absence of QVD, cells were harvested cells and resuspended in Dulbecco’s phosphate-buffered saline (DPBS). Then, cold 70% ethanol was added drop by drop with continuous mixing, followed by incubation for at least 60 min at –20 °C. The cells were collected and resuspended in DPBS. Next, propidium iodide (50 µg/ml) and RNase A (100 µg/ml) were added to the cells and incubated for 15 min. Then, the cells were analyzed using a FACS Canto II flow cytometer (BD Bioscience, USA).

### Measurement of caspase-2 and caspase-3 activity

Caspase-2 and caspase-3 activities were assessed by detecting the cleavage of fluorogenic peptide substrates Ac-VDVAD-AMC (PeptaNova, Germany) and Ac-DEVD-AFC (BD Bioscience), respectively. Harvested cells were resuspended in PBS (100 μl PBS per 1 × 10^6^ cells). Then, 25 μl of the suspension was placed in the well of a 96-well plate and mixed with the appropriate peptide substrate (100 μM) dissolved in 50 μl of caspase reaction buffer (100 mM MES, pH 6.5, 10% polyethylene glycol, 5 mM DTT, 0.001% NP-40, 0.1% CHAPS). Cleavage of the fluorogenic peptides was monitored at 37 °C using a VarioScan Flash multimode detector (Thermo Scientific) by AMC liberation at an excitation wavelength of 380 nm and an emission wavelength of 460 nm for caspase-2 and by AFC liberation at an excitation wavelength of 400 nm and an emission wavelength of 505 nm for caspase-3. The fluorescence values were normalized to protein concentrations measured using the Pierce BCA Protein Assay Kit (Thermo Scientific).

### Pull-down assay

Constructs were transfected into cells and cultured for 24–48 h. Cells were harvested by trypsinization and washed with PBS, then lysed in co-immunoprecipitation lysis buffer (10 mM Tris-HCl, pH 7.4, containing 100 mM NaCl, 1 mM EDTA, 10% glycerol, 1% Triton X-100, 1% PMSF, protease inhibitor cocktail [Roche, Switzerland], and 1 mM NEM if ubiquitin detection was necessary). Ten percent of the total lysate was analyzed by western blotting; the remainder (90%) was used for the pull-down assay. Here, 50 μl of Strep-Tactin®XT Superflow (IBA Lifesciences) was added to the 1 ml lysates with a total protein concentration of 3–5 mg/ml and incubated with gentle rotation for 30 min at 4 °C. The samples were subjected to centrifugation (3000 rpm, 3 min, 4 °C). The Strep-Tactin®XT beads were washed two times with co-immunoprecipitation lysis buffer without protease inhibitors and two times with PBS. Then, the beads were dried with a syringe. Twenty microlitres of 2× Laemmli buffer was added, and the samples were incubated for 5 min at 95 °C.

### MS analysis

#### Immunoprecipitation and sample preparation

Cells were transfected with the appropriate constructs and cultured for 24–48 h. Cells were harvested by trypsinization, washed with PBS, and lysed in immunoprecipitation lysis buffer (20 mM Tris-HCl (pH 7.4), 135 mM NaCl, 2 mM EDTA, 10% glycerin, 1% Triton X-100, 1 mM PMSF, and 1% protease inhibitor cocktail [Roche]). Cell lysates were cleared by centrifugation at 4 °C. Equal amounts of total protein from each sample were incubated with a rabbit anti-caspase-2L polyclonal antibody (C-20, Santa Cruz Biotechnology, USA) for 2 h at 4 °C. Then protein A Sepharose beads (Sigma-Aldrich) were added, and the samples were incubated with gentle rotation overnight at 4 °C. The beads were washed two times with immunoprecipitation lysis buffer and two times with PBS containing protease inhibitors. Protein A Sepharose beads with bound proteins were dried with a syringe, washed three times with 50 mM ammonium bicarbonate, and digested with 0.4 µg trypsin in 100 µl of DPBS overnight at 37 °C. The supernatant was removed from the beads, and the beads were washed with fresh ammonium bicarbonate and pooled together. The samples were desalted on 18 C pipette tips (Thermo Scientific) according to the manufacturer’s instructions; the obtained peptides were dried and then dissolved in 0.1% (v/v) formic acid in water before being used for liquid chromatography-tandem mass spectrometry (LC-MS/MS).

#### LC-MS/MS

Automated online analyses were performed with an LTQ Orbitrap Velos Pro hybrid mass spectrometer (Thermo Scientific, USA) equipped with a nano-electrospray source. Online reversed-phase LC to separate peptides was performed using an EASY-nLC 1200 system (Thermo Scientific). All samples were analyzed on a 20 mm × 100 μm C18 pre-column followed by a 100 mm × 75 μm C18 column with a particle size of 5 μm (NanoSeparatoons, the Netherlands) at a flow rate of 300 nl/min with an EASY-nLC II system (Thermo Scientific) using a gradient of Buffer A (0.1% formic acid) and Buffer B (0.1% formic acid in acetonitrile) at a flow rate of 300 nl/min as follows: from 2% B to 40% B in 90 min; 40% B to 90% B in 20 min; and 90% B for 10 min. Full scans were performed at a resolution of 60,000; the top 20 most intense multiply charged ions were selected with an isolation window of 2.0 and fragmented in the linear ion trap by collision-induced dissociation with a normalized collision energy of 30%. Dynamic exclusion of sequenced peptides for 60 s and charge state-filtering disqualifying singly charged peptides was activated and predictive automatic gain control (AGC) was enabled. The histograms illustrating peptide number and peptide length distribution were made using GraphPad Prism 8.0 (GraphPad Software, USA).

### Sodium dodecyl sulfate-polyacrylamide gel electrophoresis (SDS-PAGE) and western blotting

Laemmli loading buffer was mixed with 30 μg of protein from cell lysates. After boiling for 5 min at 95 °C, the samples were subjected to SDS-PAGE. The gels consisted of a 12% acrylamide separation gel and a 4% stacking gel. After electrophoresis, the separated protein was transferred to nitrocellulose membranes using the BioRad TransBlot Turbo apparatus (Bio-Rad). Then, the membranes were incubated in 5% non-fat milk in PBST (PBS + 0.05% Tween-20) and washed in PBST for 3 × 10 min. The membranes were incubated with the following primary antibodies overnight at 4 °C: caspase-2 (611022, BD Transduction Lab, USA), caspase-3 (9661 T and 9662S), p62 (8025S), GAPDH (2118 L), K48-linkage specific polyubiquitin (12805S) (all from Cell Signalling, USA), tubulin (ab7291), PARP (ab137653), vinculin (ab129002) (all from Abcam, USA), RAIDD (ALX8210-915-C050), PIDD1 (ALX804-837-C100) (both from Enzo Life Sciences, UK), and ubiquitin (NB300-130, Novus Biologicals, USA). After three washes in PBST, the membranes were incubated with goat anti-rabbit IgG conjugated to horseradish peroxidase (ab97200) or goat anti-mouse IgG conjugated to horseradish peroxidase (ab97265, both from Abcam) at RT for 1 h. Then, the membranes were washed four times in PBST and incubated with ECL (Promega, USA) or SuperSignal West Dura Extended Duration Substrate (Thermo Scientific) to visualize protein bands. Images were acquired with the Imager ChemiDoc™ (Bio-Rad).

### BiFC

The constructs encoding Casp2-CARD VN and Casp2-CARD were kindly provided by Professor Lisa Bouchier-Hayes (Baylor College of Medicine, Houston, TX, USA). Cells were transfected with Casp2-CARD VN (20 ng) and Casp2-CARD VC (20 ng) as described previously [30], along with 500 ng of expression plasmid encoding wild type or mutant p62 fused to mCherry. On the next day, transfected cells were imaged using an LSM 780 confocal laser scanner microscope (Zeiss, Germany).

### Cox regression analysis

Cox regression analysis was performed using OncoLnc (http://www.oncolnc.org/).

### Data processing and statistical analysis

Western blot images were processed using Image Lab software (Bio-Rad, USA). Densitometric analysis was performed using Image Lab Software or Image J (National Institutes of Health, USA). For normally distributed data, a Student’s *t*-test was performed to analyze statistically significant differences between the groups. A *p* value < 0.05 was considered to be statistically significant.

## Supplementary information

Supplementary material

Raw Western blot data

## Data Availability

All data supporting this study are presented in this published article and in its Supplementary information files.
