# Supplementary material for: Ally or traitor: the dual role of p62 in caspase-2 regulation
Source: Cell Death Dis. 2024 Nov 14;15(11):827. doi: 10.1038/s41419-024-07230-3 (PMC11564777; doi:10.1038/s41419-024-07230-3)
Supplement: Supplementary file 1 — Supplementary material [file 41419_2024_7230_MOESM1_ESM.docx]

Supplementary materials

**Ally or traitor: the dual role of p62 in caspase-2 regulation**

Pavel I. Volik^1,2^, Alexey V. Zamaraev^1,2^, Aleksandra Y. Egorshina^1^, Nikolay V. Pervushin^1,2^, Anastasia A. Kapusta^2^, Pyotr A. Tyurin-Kuzmin^2^, Anastasia V. Lipatova^1^, Thilo Kaehne^4^, Inna N. Lavrik^4^, Boris Zhivotovsky^1,2,3*^ and Gelina S. Kopeina^1,2,*^

^1^Engelhardt Institute of Molecular Biology, RAS, 119991, Moscow Russia;

^2^Faculty of Medicine, MV Lomonosov Moscow State University, 119991 Moscow, Russia;

^3^Division of Toxicology, Institute of Environmental Medicine, Karolinska Institutet, Box 210, 17177 Stockholm, Sweden;

^4^Translational Inflammation Research, Medical Faculty, Center of Dynamic Systems (CDS), Otto von Guericke University, 39106, Magdeburg, Germany.

*Correspondence should be sent to:

Boris Zhivotovsky ([boris.zhivotovsky@ki.se](mailto:boris.zhivotovsky@ki.se)), Gelina Kopeina ([lirroster@gmail.com](mailto:lirroster@gmail.com))


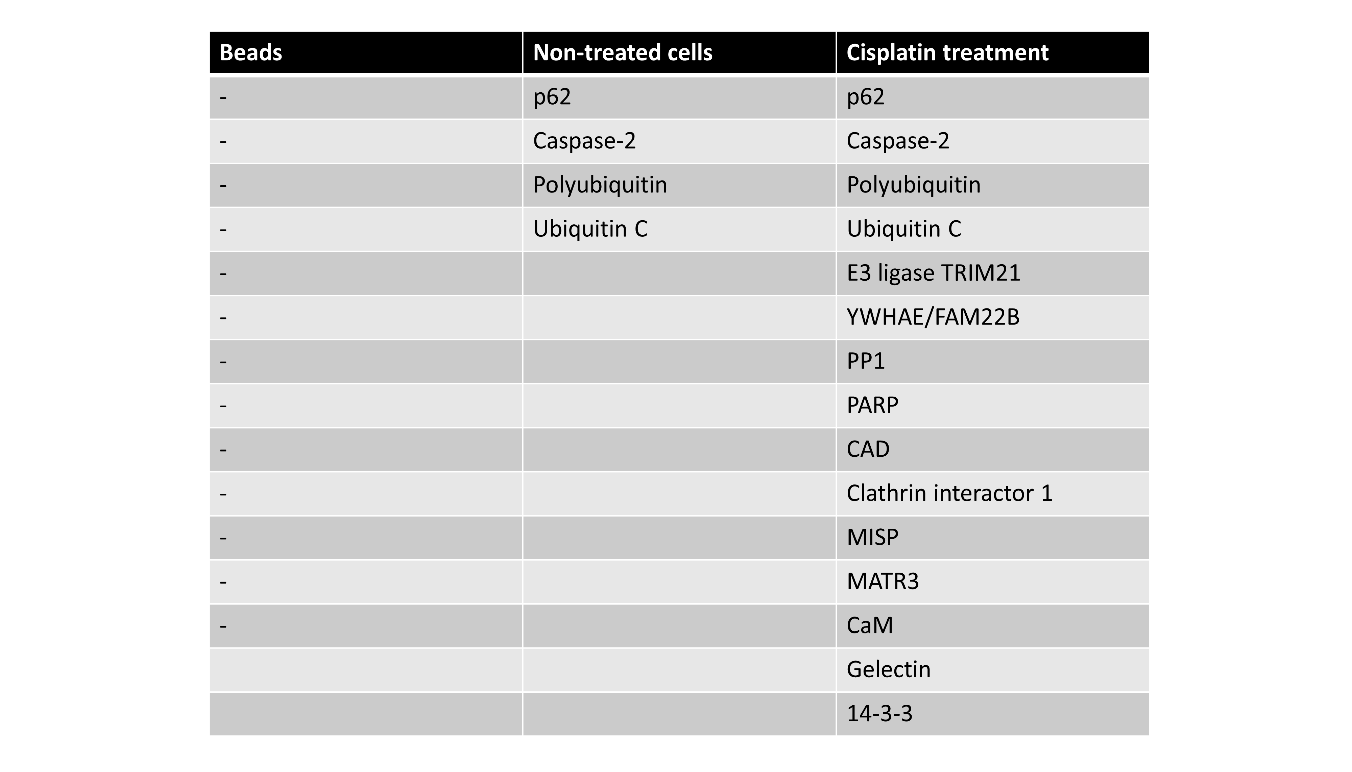


**Fig S1.** The list of proteins co-precipitated with caspase-2 in normal conditions and after DNA damage induction. Abbreviations: PP1, Protein phosphatase 1; PARP, Poly (ADP-ribose) polymerase; CAD, Caspase-activated DNase; MISP, Mitotic interactor and substrate of PLK1; MATR3, Matrin-3; CaM, Calmodulin.


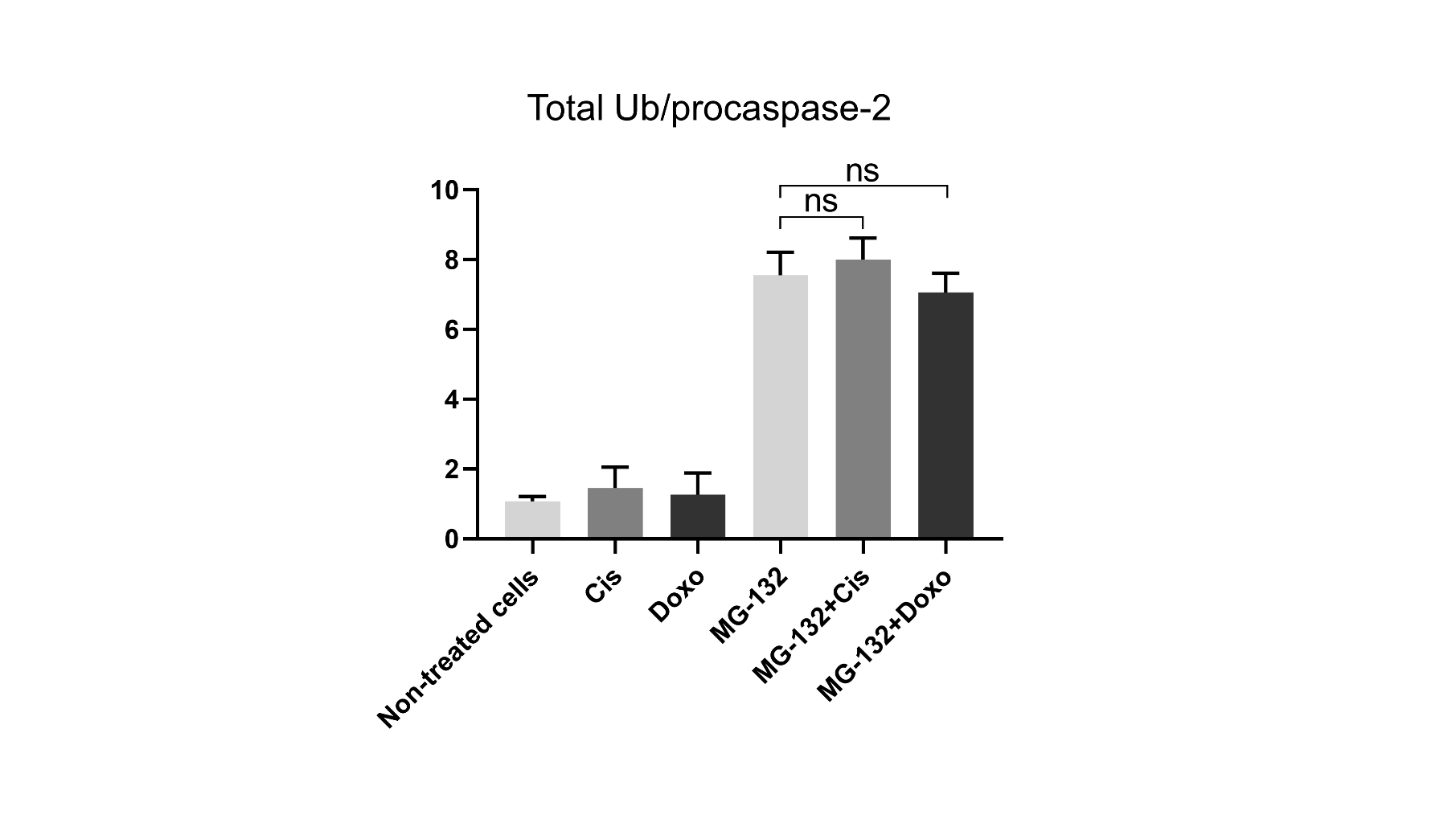


**Fig S2.** Densitometry for western blot (pull down) shown in Fig. 1E. Data are representative of three independent experiments and values are expressed in mean ± SEM.


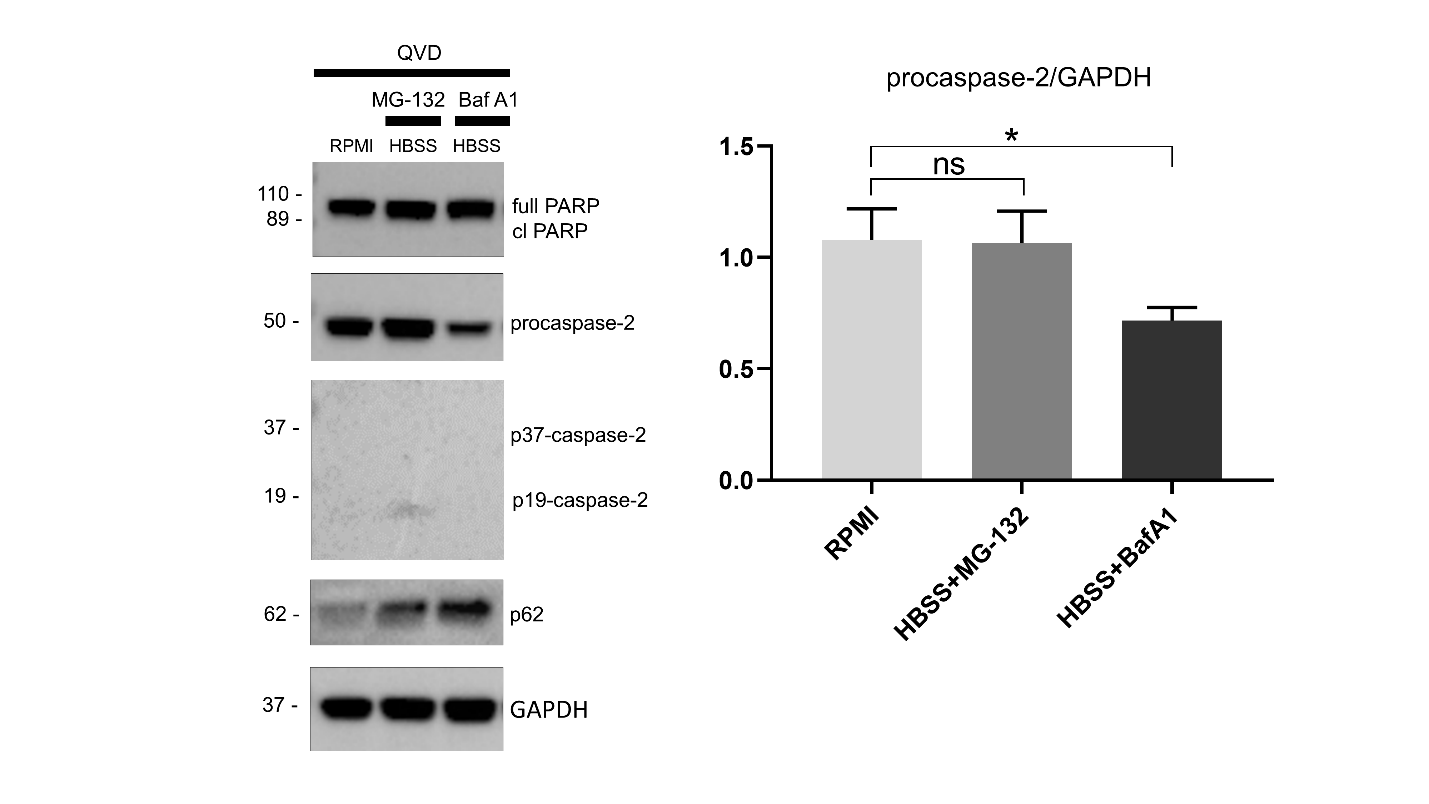


**Fig S3.** Nutrient limitation-induced procaspase-2 degradation in HEK 293T cells. After growing in Roswell Park Memorial Institute 1640 (RPMI 1640) medium or Hank's balanced salt solution (HBSS) medium, cells were treated with 25 nM bafilomycin A1 (BafA1) for 18 h or 1 µm MG-132 for 6 h. Cell death was estimated by an accumulation of cleaved poly (ADP-ribose) polymerase (cl PARP). To suppress caspase activation and apoptosis induction, 25 µm QVD were used. One representative experiment from three independent experiments is shown. Abbreviations: GAPDH, Glyceraldehyde 3-phosphate dehydrogenase.


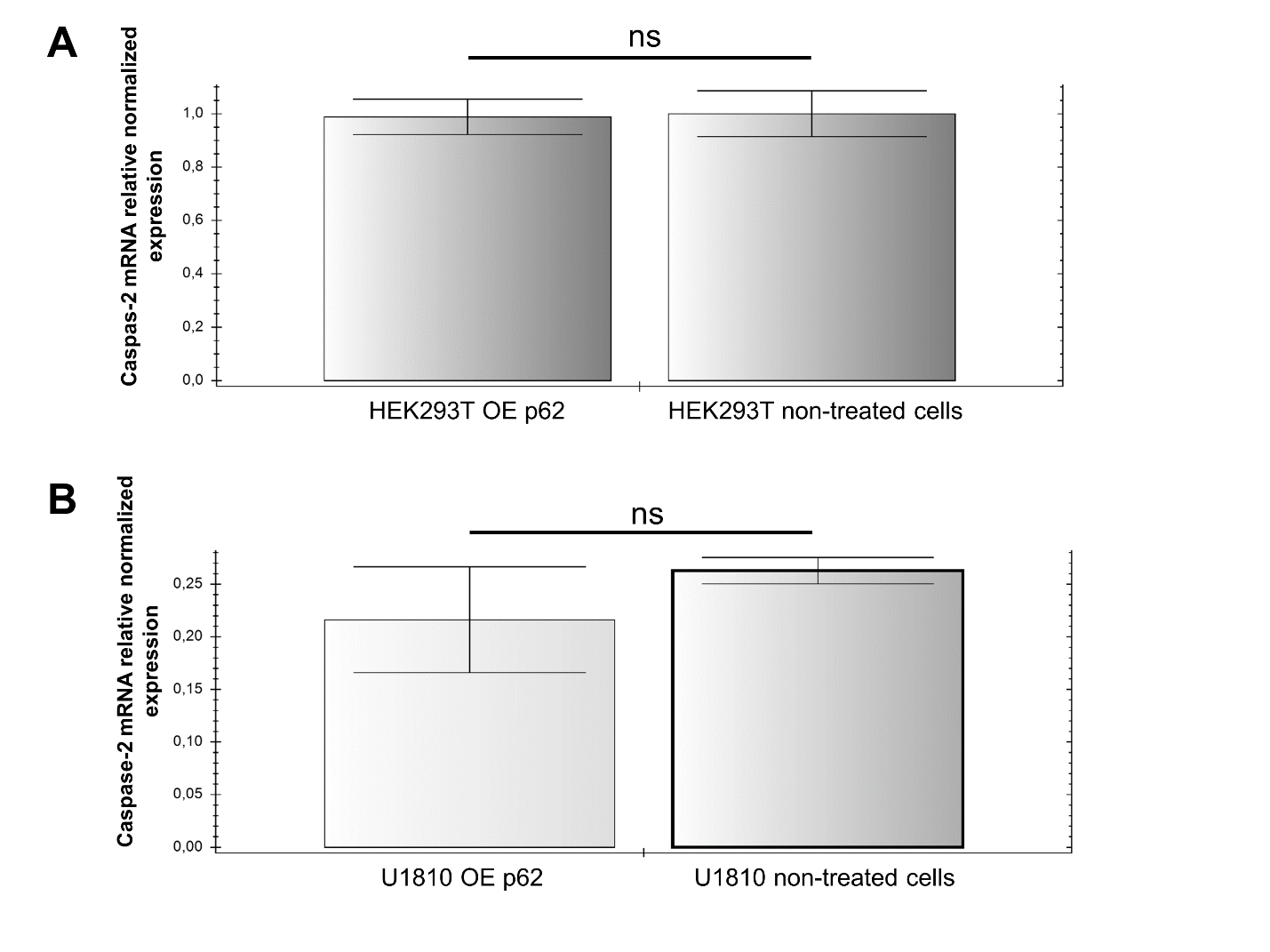


**Fig S4.** Overexpression of p62 does not affect caspase-2 mRNA levels in HEK 293T (**A**) and U1810 (**B**) cells. qPCR data were obtained from at least three independent experiments. Data are presented as mean ± SD (error bars).


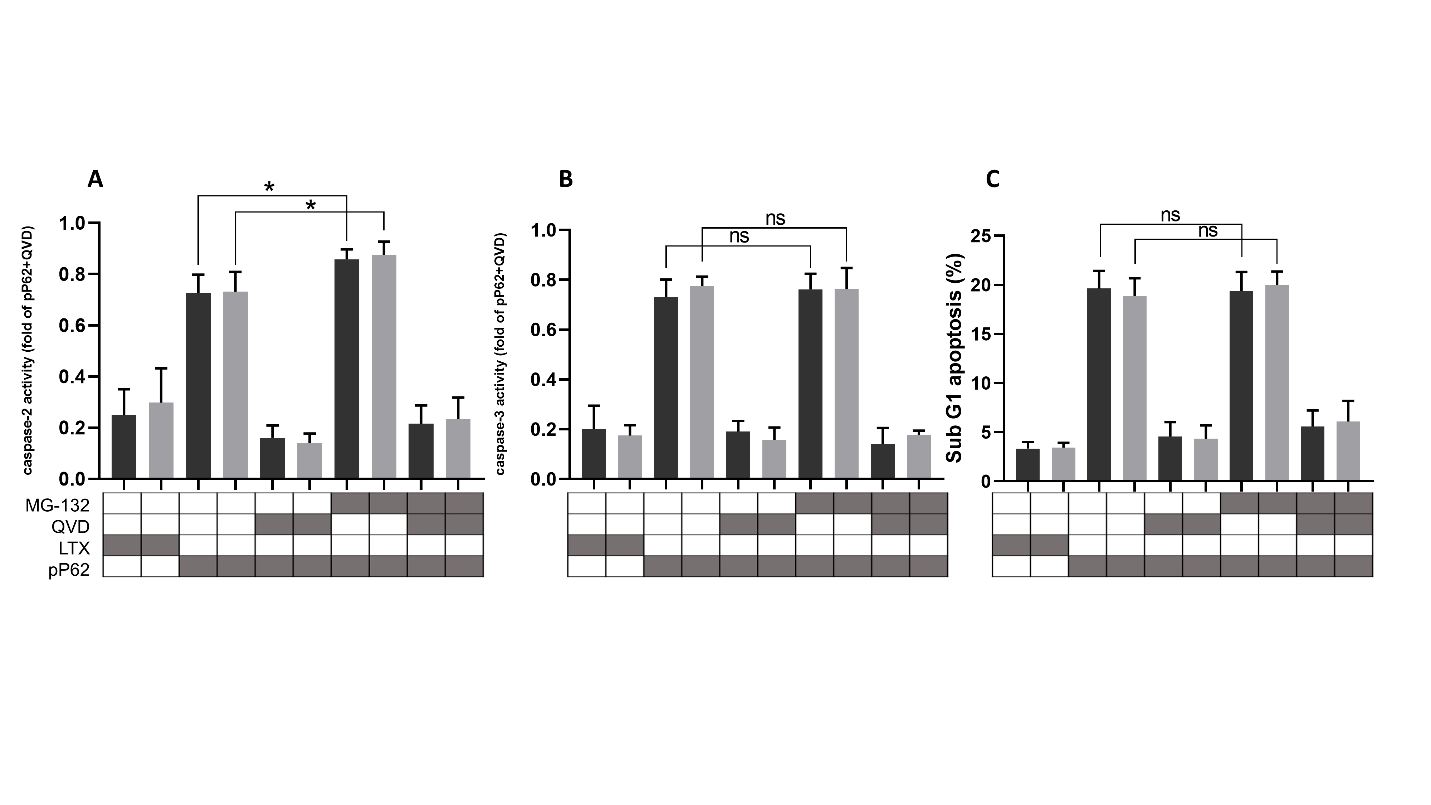
**Fig S5.** Overexpression of p62 leads to increased caspase-2 (**A**) and caspase-3 (**B**) peptidase activities in the HEK293T cells. Caspase-2 and caspase-3 activities were measured using substrates Ac-VDVAD-AMC and Ac-DEVD-AFC, respectively, in lysates of cells with overproduction of p62. Cells were treated with 1 µm MG-132 for 6h and/or 20 µm QVD for 24 h and caspase activity was measured. The histograms demonstrate the slope of the enzymatic activity curves. (**C**) Transient expression of p62 induces apoptosis in HEK293T cells. SubG1 apoptotic peak was determined by flow cytometry. A graphical presentation of the percentage of the SubG1 peak in apoptotic cells is shown. Results are presented as the mean of three distinct experiments ± SEM.


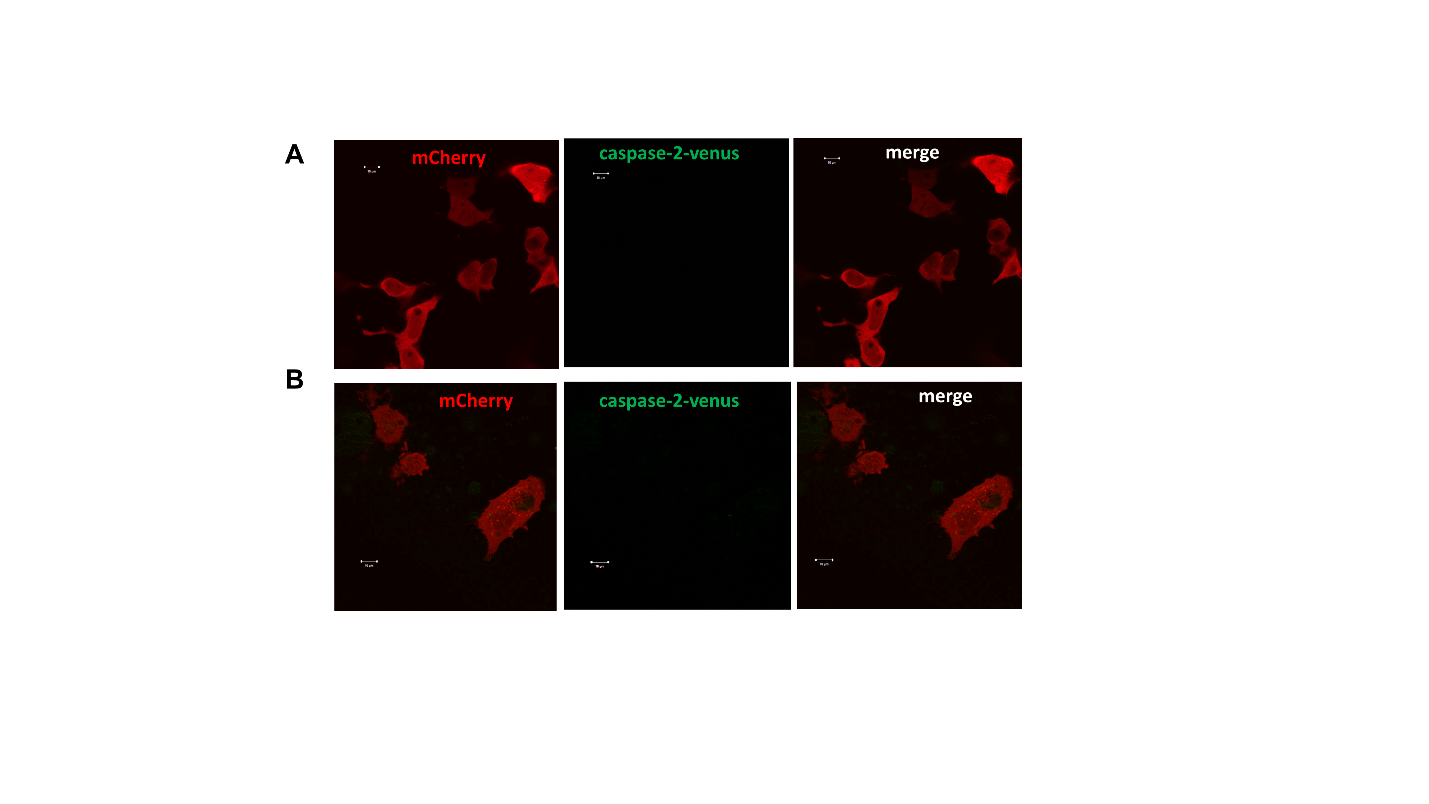
**Fig S6.** Overexpression of mCherry does not result in Venus fluorescence. HEK293T (**A**) and U1810 (**B**) cells were transiently transfected with Casp2-CARD VN and Casp2-CARD VC along with expression plasmid encoding mCherry. Representative images of cells after 24h transfection are shown. Venus-positive foci are *green*, mCherry – *red*. Scale bars represent 20μm.

**
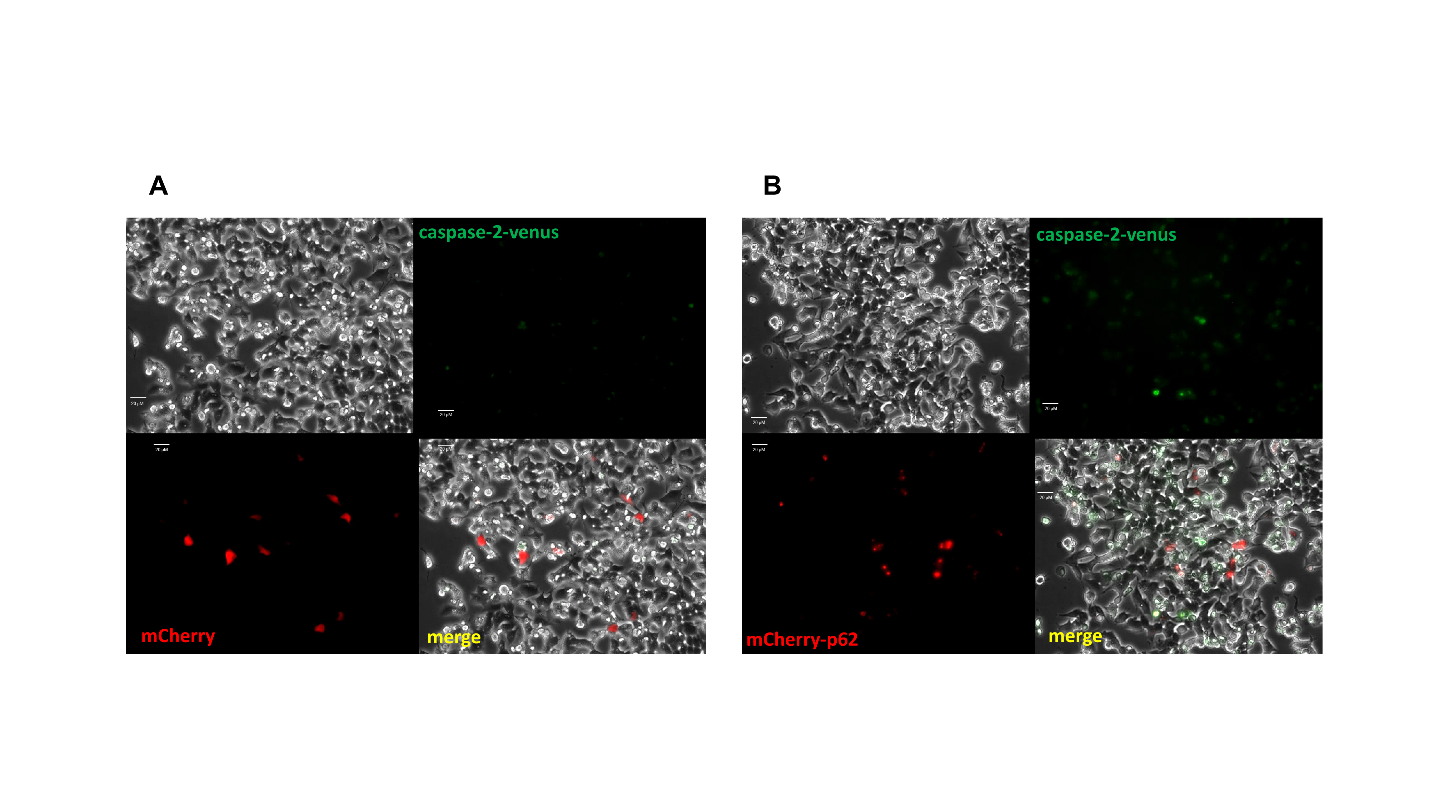
Fig S7.** Overexpression of p62 induces dimerization of caspase-2 in a caspase-8-independent manner. Caspase-8-silenced SK-N-BE cells were transiently transfected with Casp2-CARD VN and Casp2-CARD VC along with expression plasmids encoding mCherry (**A**) or mCherry-p62 (**B**). Representative images of cells after 24h transfection are shown. Venus-positive foci are *green*, mCherry/mCherry-p62 – *red*. Scale bars represent 20μm.


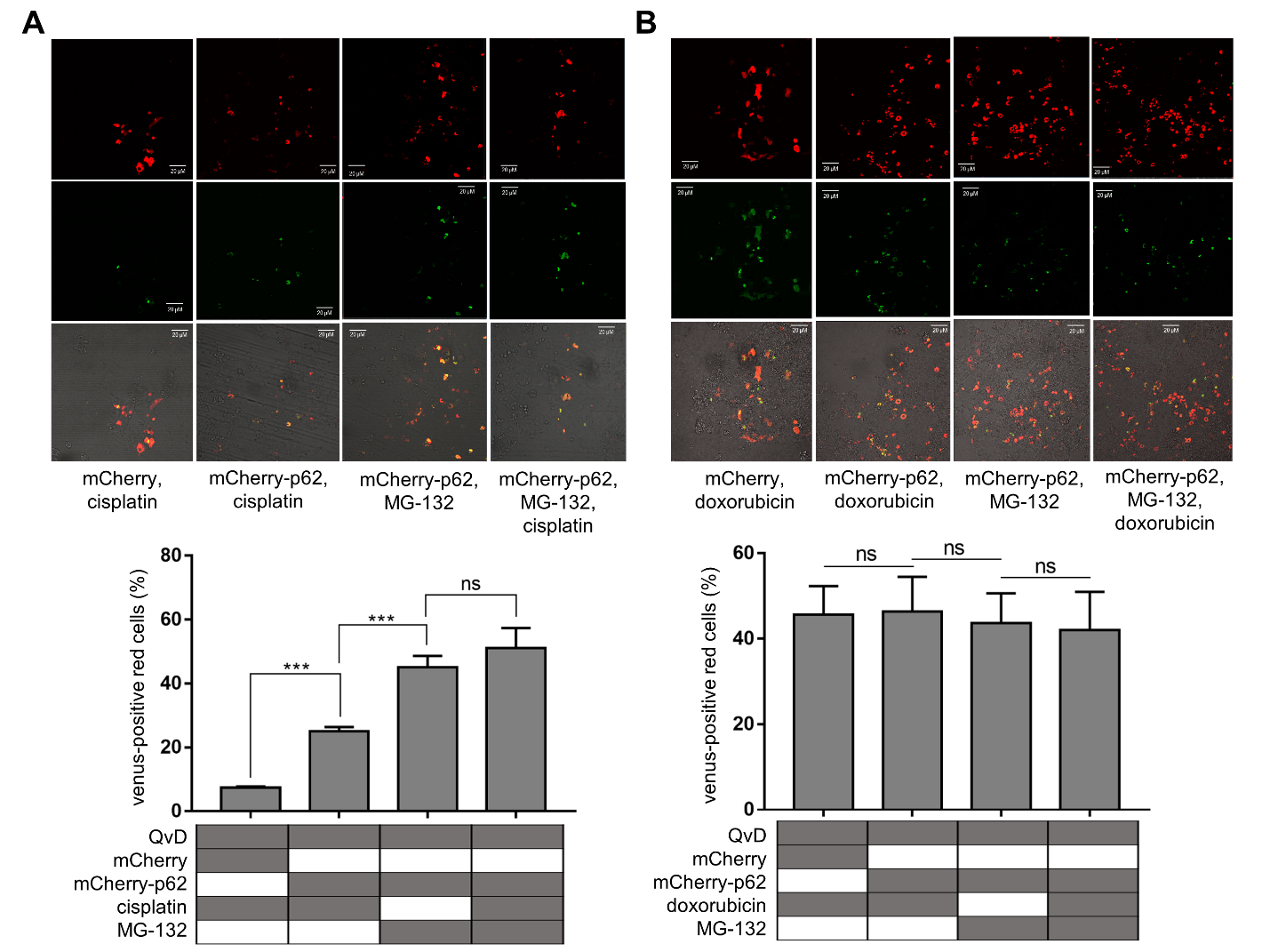
**Fig S8.** Caspase-2 dimerization in response to DNA damage and proteasome inhibition. HEK293T cells were transfected with Casp2-CARD VN (20ng) and Casp2-CARD VC (20ng), along with 500ng of expression plasmid encoding mCherry-p62 in several probes. MG-132 was used for proteasome inhibition. Cell death was inhibited by QVD. DNA damage-induced cells were treated with 25 µM cisplatin (**A**) and 2 µM doxorubicin (**B**) for 18 h. Representative confocal images of cells are shown. Scale bars represent 50μm. The percentage of mCherry-p62-positive (*red*) cells that were Venus-positive (*green*) was determined. Results represent triplicate counts with error bars representing standard deviation.


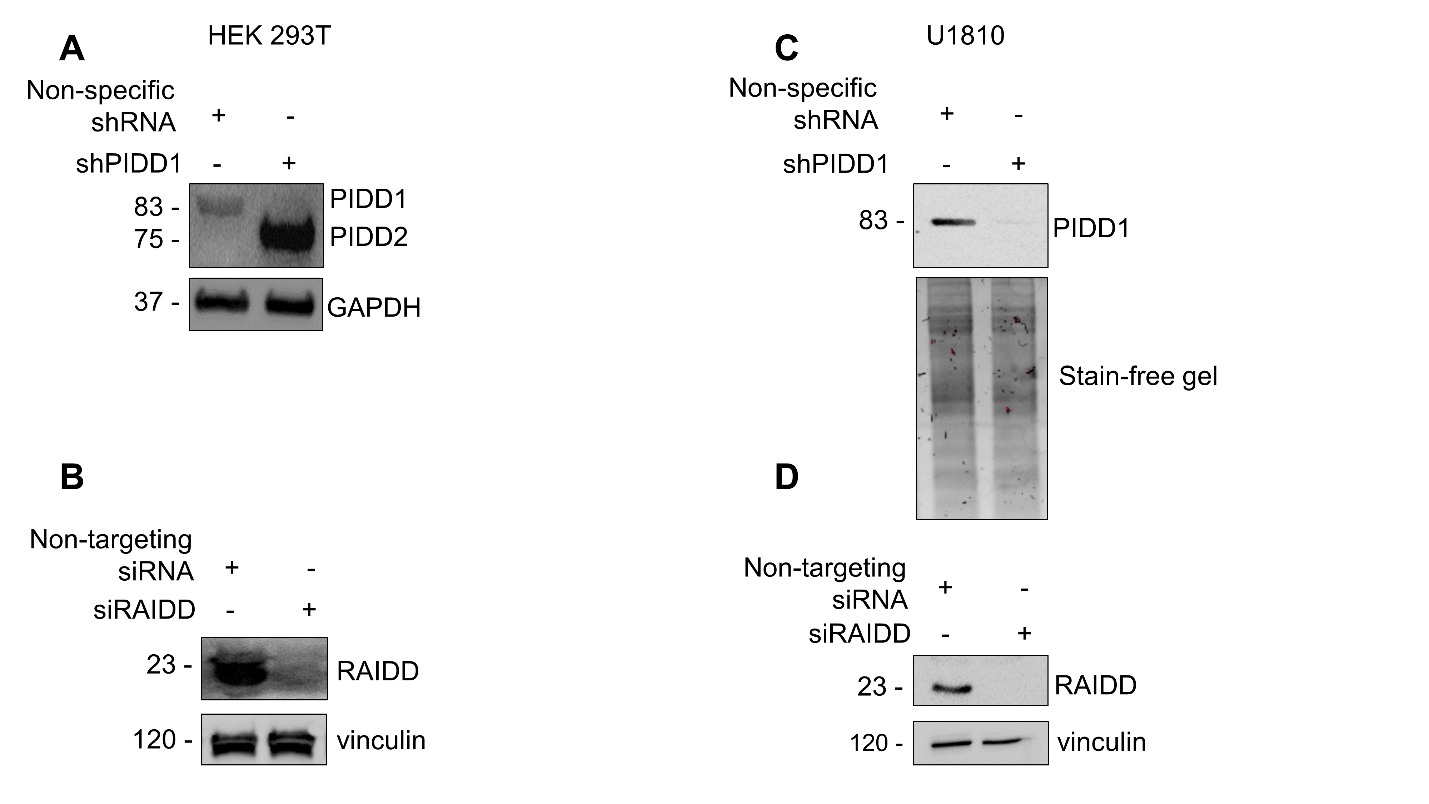
**Fig S9.** Suppression of expression of PIDDosome components with shRNA and siRNA. Western blot analysis of lysates of HEK293T (**A**) and U1810 (**C**) cells stably expressing shPIDD1. Cells expressing non-specific shRNA were used as a negative control. To inhibit RAIDD expression, HEK293T (**B**) and U1810 (**D**) cells were transfected with siRAIDD. Non-targeting siRNA was used as a negative control.


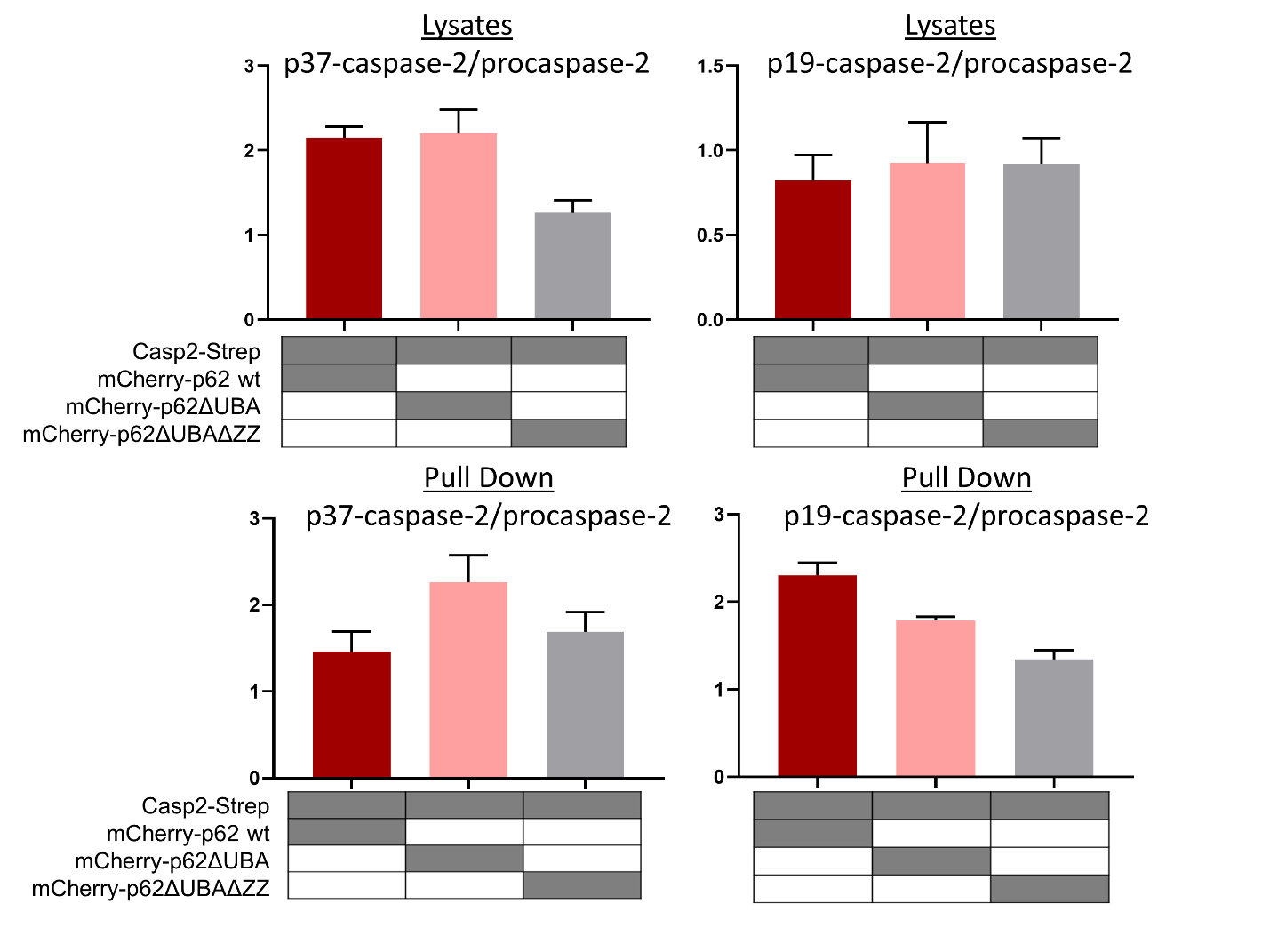
**Fig S10.** Densitometry of western blots (pull down and lysates) with U1810 samples shown in Fig. 7). Data are representative of three independent experiments and values are expressed in mean ± SEM.


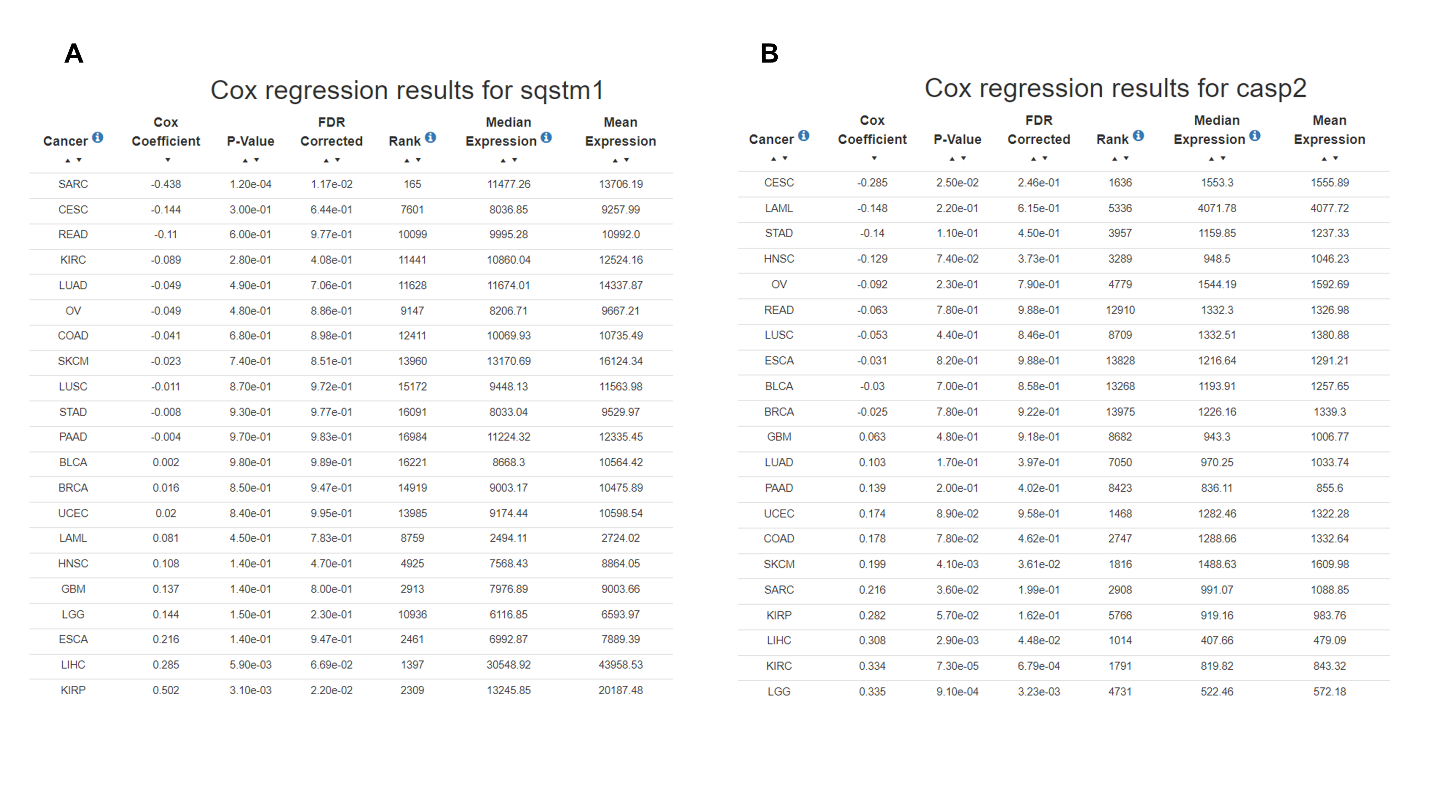
**Fig S11.** Cox regression analysis of SQSTM1 (**A**) and Casp2 (**B**) in different types of cancers. Datasets were utilized from OncoLnc.
