# Supplementary material for: Ally or traitor: the dual role of p62 in caspase-2 regulation
Source: Cell Death Dis. 2024 Nov 14;15(11):827. doi: 10.1038/s41419-024-07230-3 (PMC11564777; doi:10.1038/s41419-024-07230-3)
Supplement: Supplementary file 2 — Raw Western blot data [file 41419_2024_7230_MOESM2_ESM.pdf]

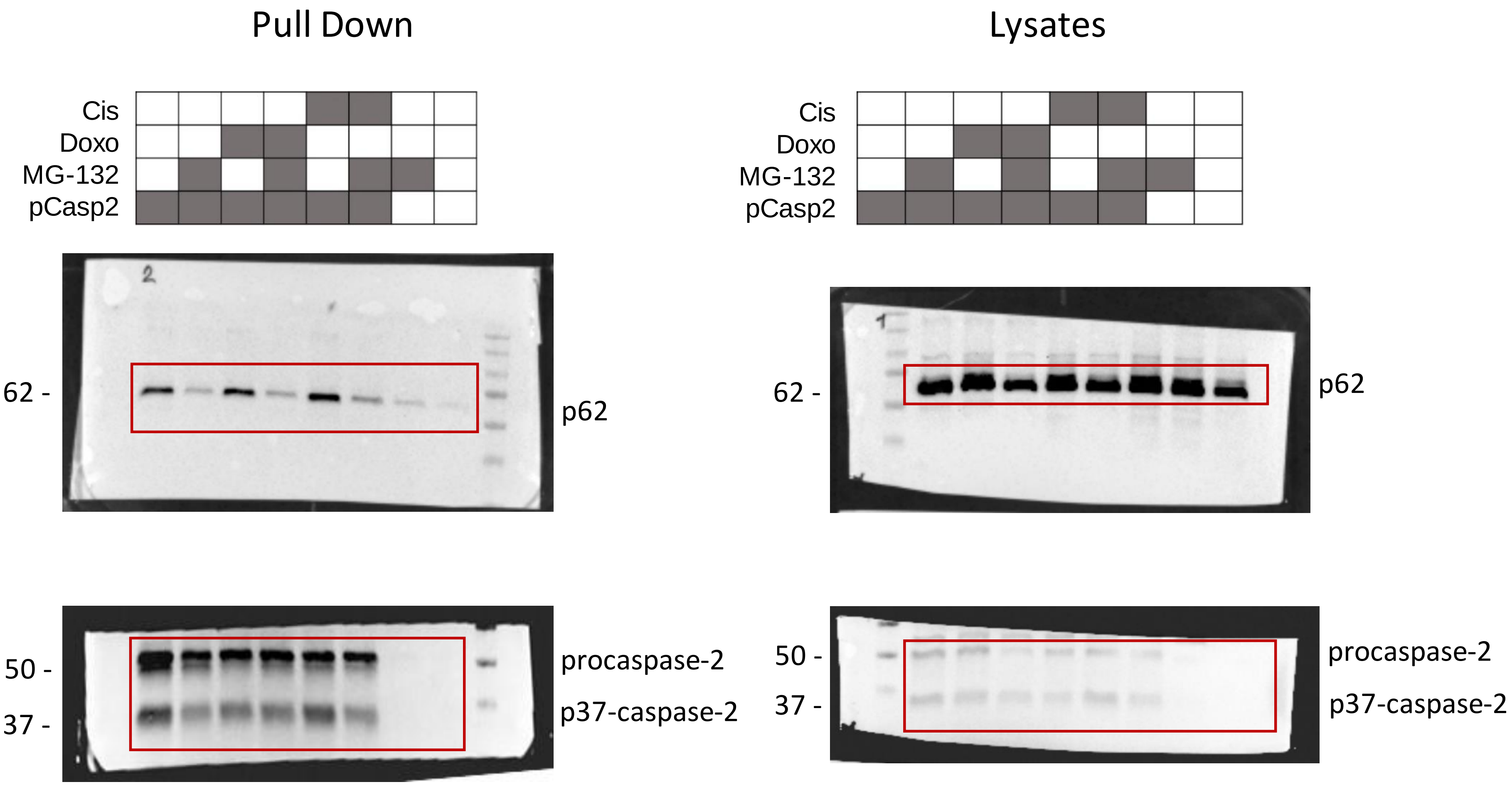
Pull Down
Lysates
Cis
Doxo
MG-132
pCasp2
62 -
p62
50 -
37 -
procaspase-2
p37-caspase-2

Fig. 1D

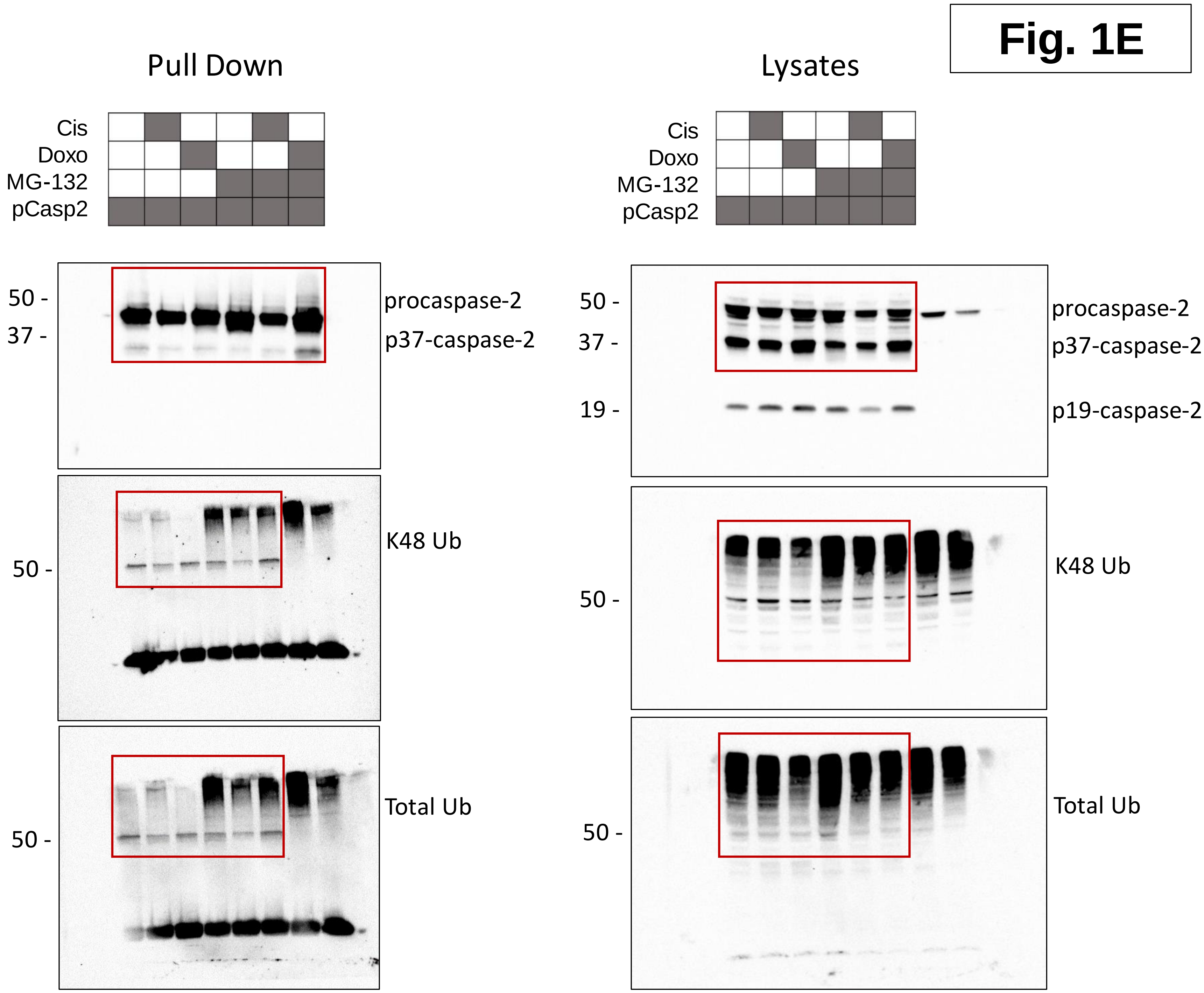
Fig. 1E
Pull Down
Lysates
Cis
Doxo
MG-132
pCasp2
50 -
37 -
19 -
procaspase-2
p37-caspase-2
p19-caspase-2
K48 Ub
Total Ub

Fig. 2A*

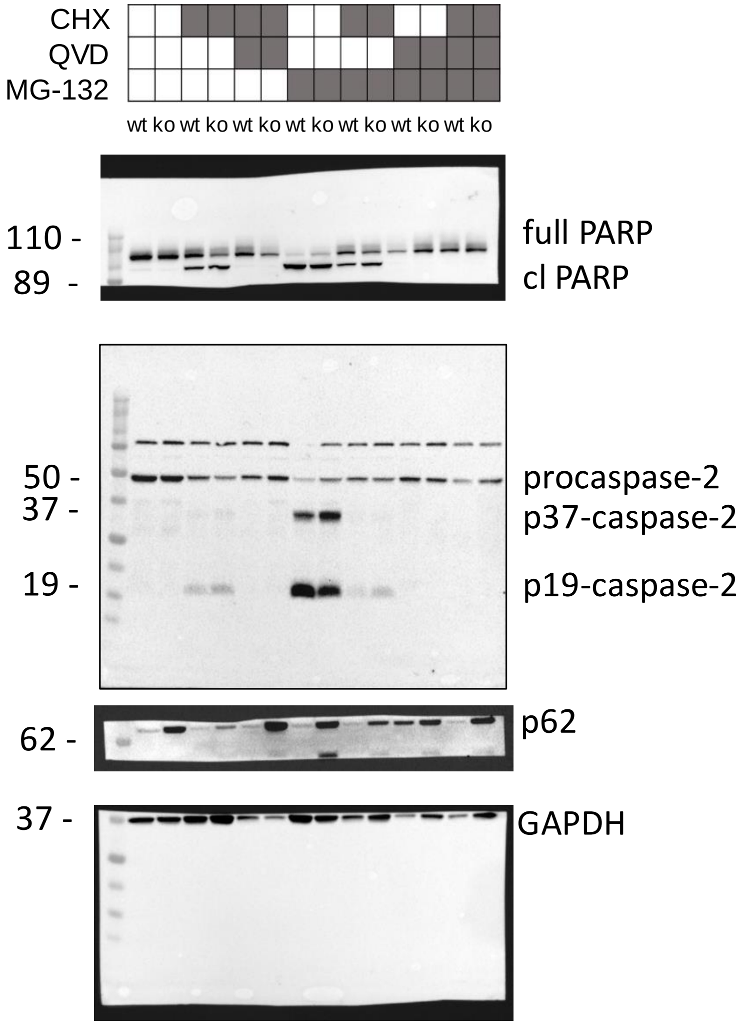

Fig. 2B

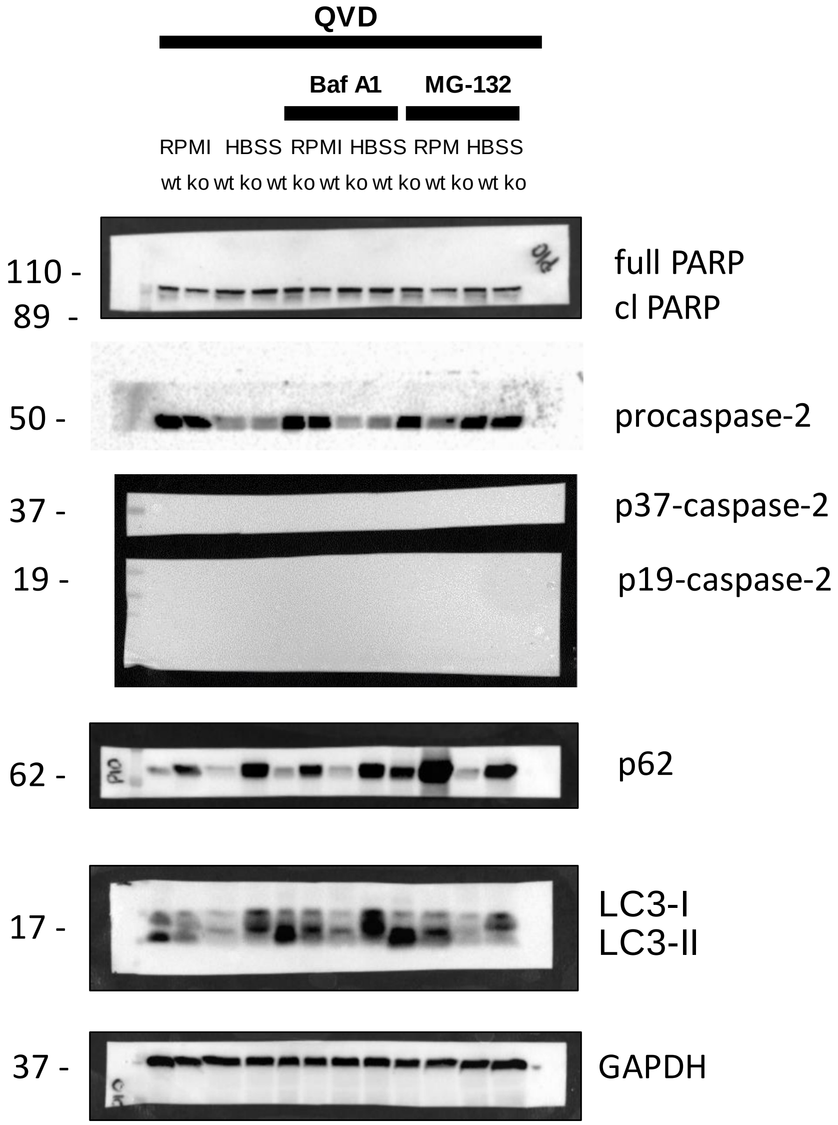

* The original representative blot has not been preserved, here is another repeat of the experiment

Fig. S3

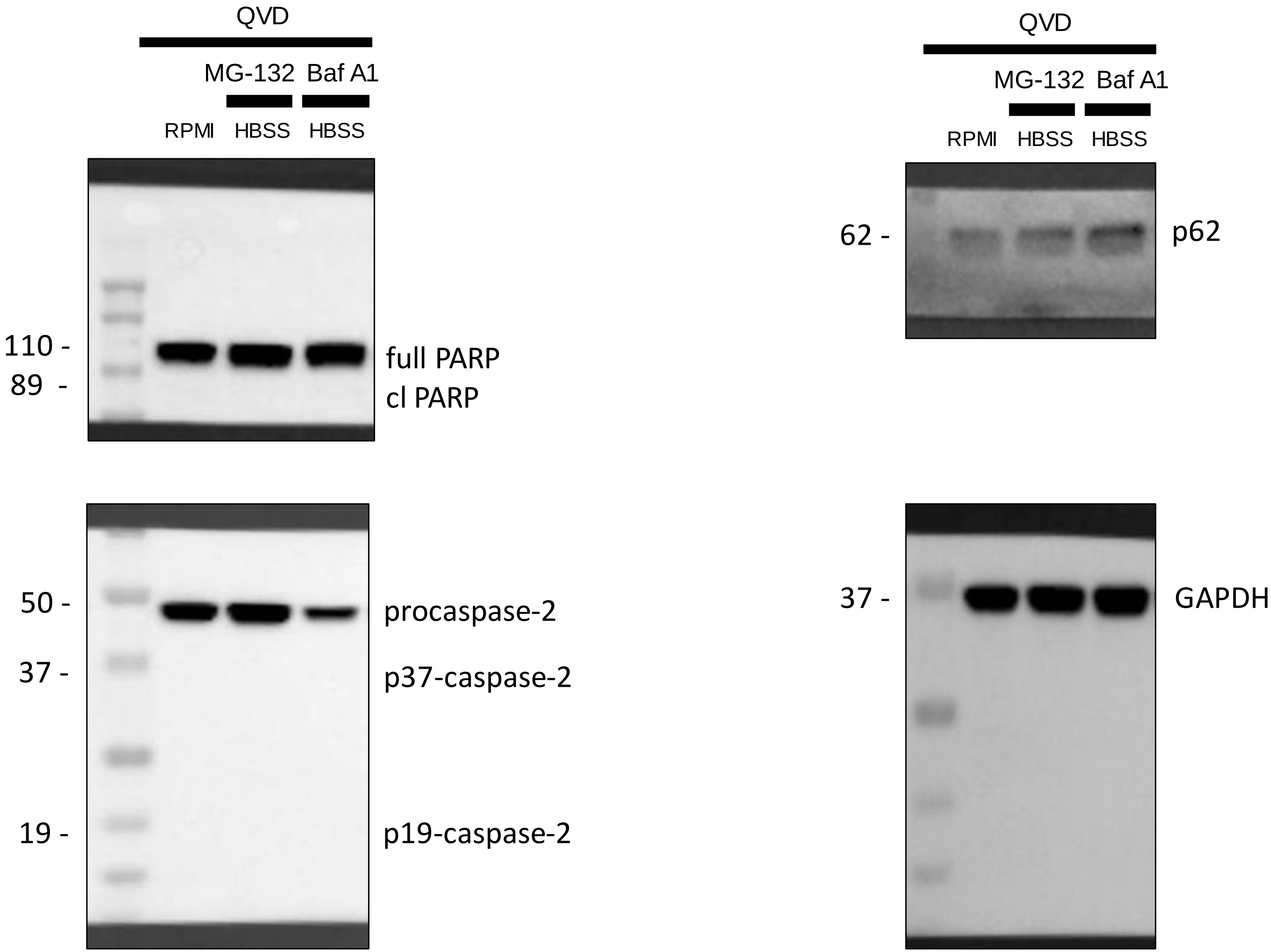
QVD
MG-132
Baf A1
RPMI
HBSS
HBSS
110 -
89 -
full PARP
cl PARP
50 -
37 -
19 -
procaspase-2
p37-caspase-2
p19-caspase-2
QVD
MG-132
Baf A1
RPMI
HBSS
HBSS
62 -
p62
37 -
GAPDH

Fig. 3A

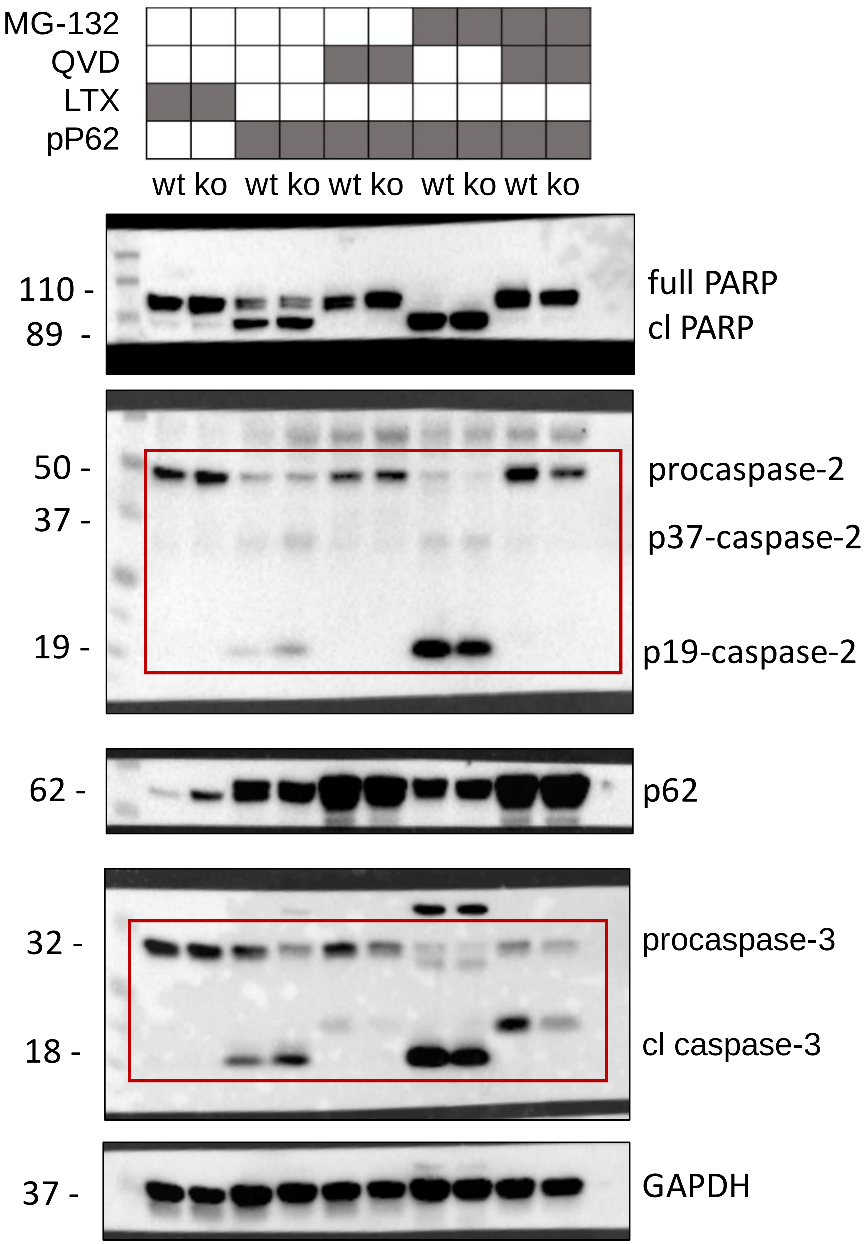
MG-132
QVD
LTX
pP62
wt ko wt ko wt ko wt ko wt ko
110 -
89 -
full PARP
cl PARP
50 -
37 -
19 -
procaspase-2
p37-caspase-2
p19-caspase-2
62 -
p62
32 -
18 -
procaspase-3
cl caspase-3
37 -
GAPDH

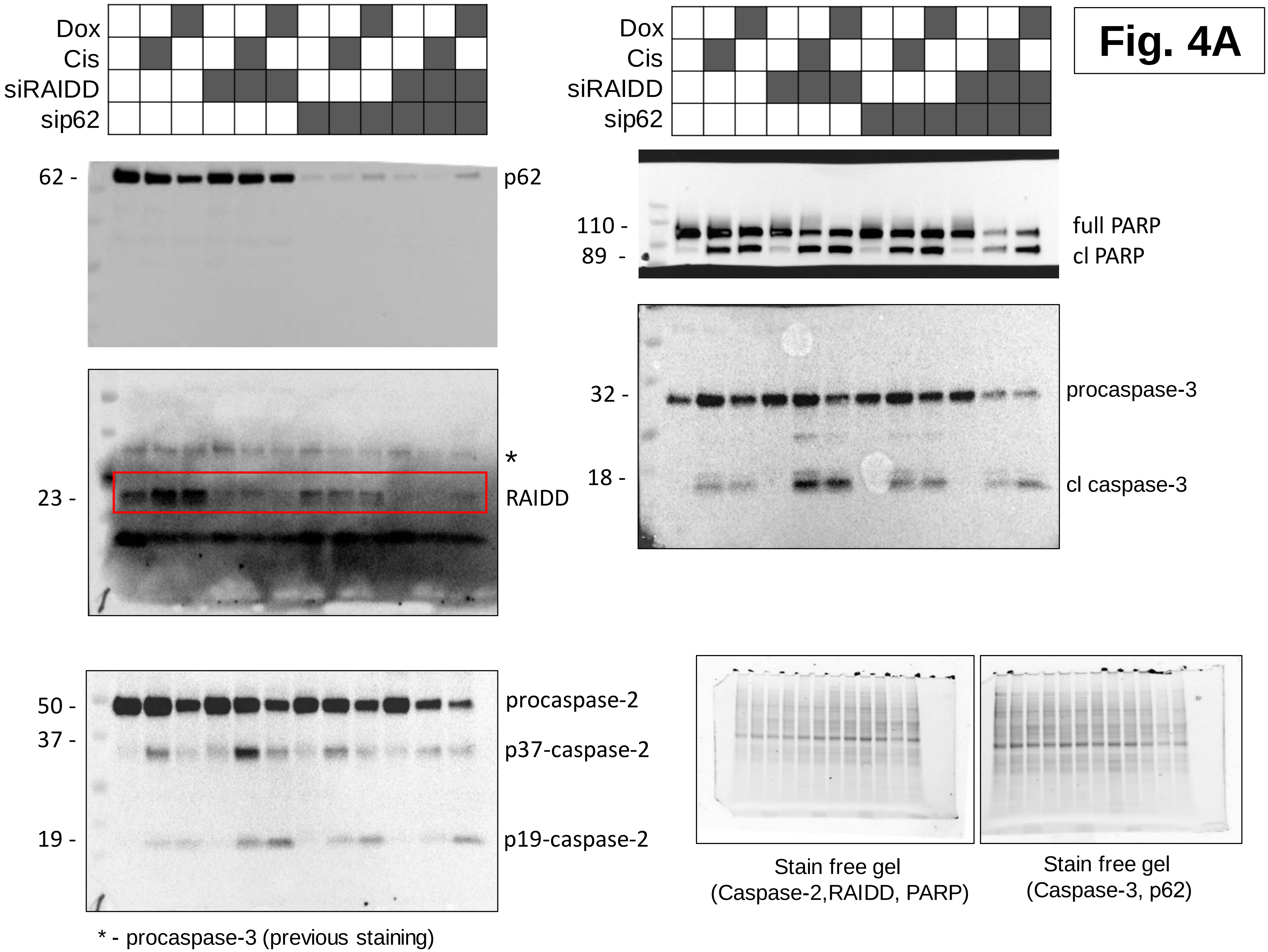

Fig. 4A
Dox
Cis
siRAIDD
sip62
62 -
p62
110 -
89 -
full PARP
cl PARP
32 -
18 -
procaspase-3
cl caspase-3
23 -
*
RAIDD
50 -
37 -
19 -
procaspase-2
p37-caspase-2
p19-caspase-2
Stain free gel
(Caspase-2,RAIDD, PARP)
Stain free gel
(Caspase-3, p62)
* - procaspase-3 (previous staining)

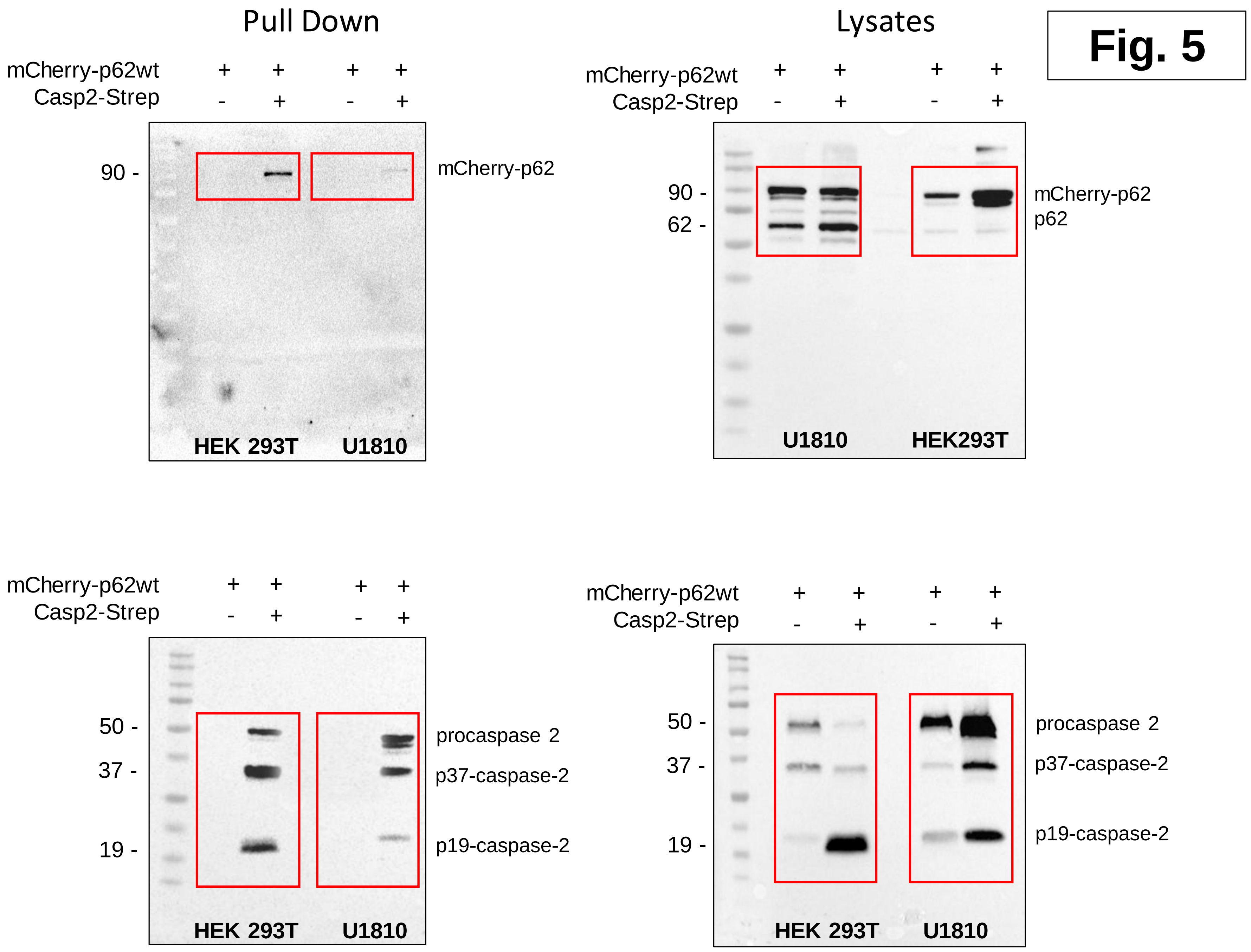
Fig. 5
Pull Down
Lysates
mCherry-p62wt
Casp2-Strep
+ + + +
- + - +
90 -
62 -
50 -
37 -
19 -
mCherry-p62
p62
procaspase 2
p37-caspase-2
p19-caspase-2
HEK 293T
U1810
HEK293T

Fig. S9A

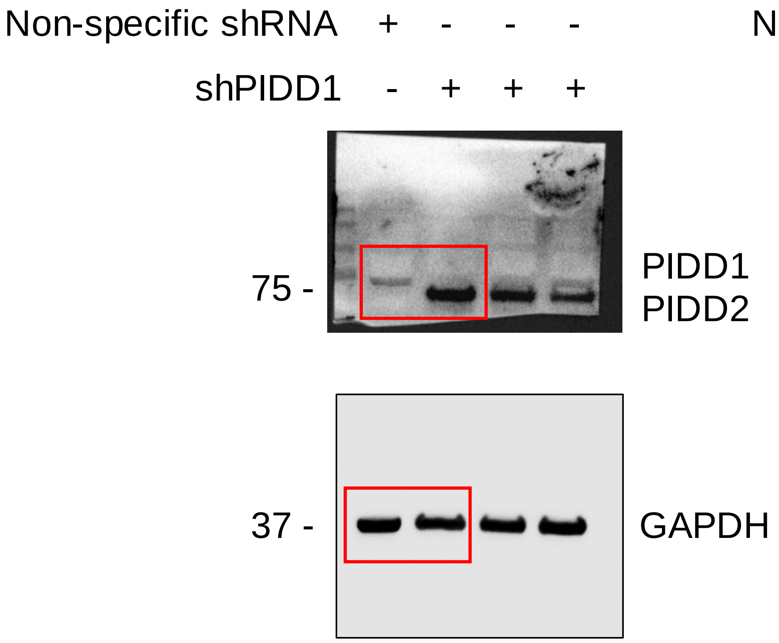

Fig. S9B

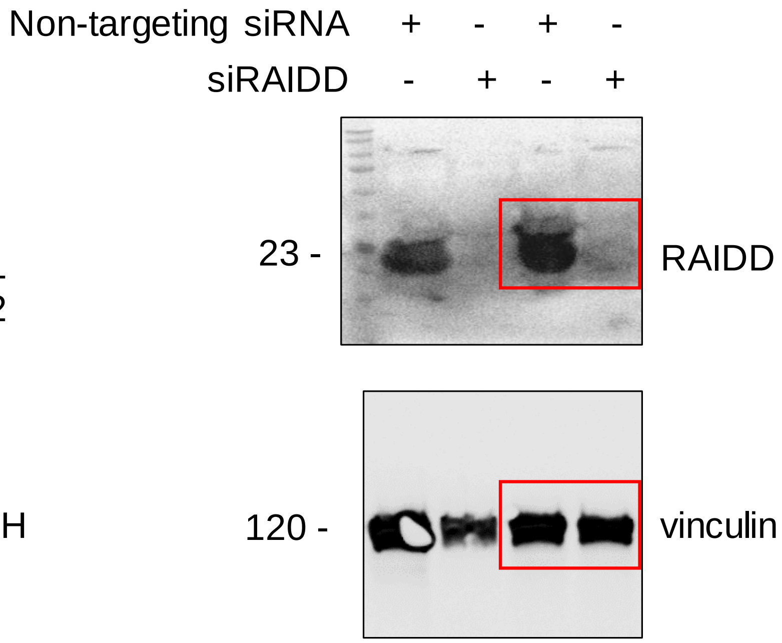

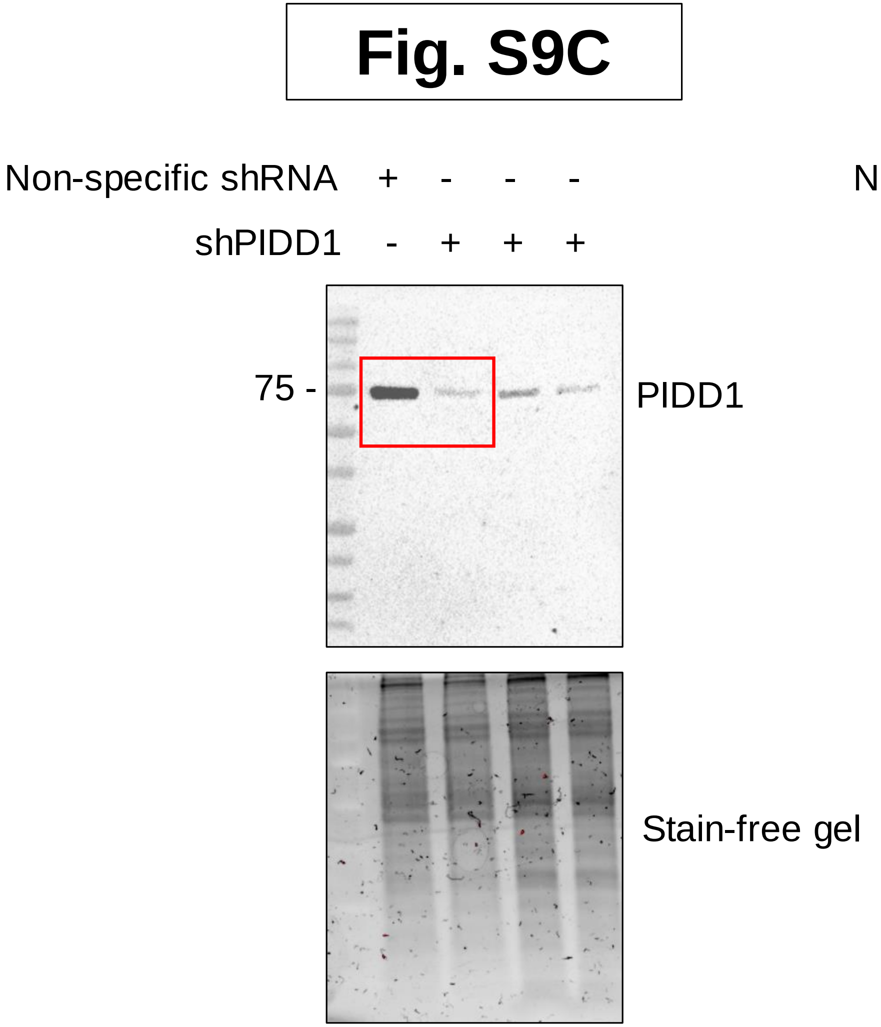
Fig. S9C
Non-specific shRNA + - - -
shPIDD1 - + + +
75 -
PIDD1
Stain-free gel

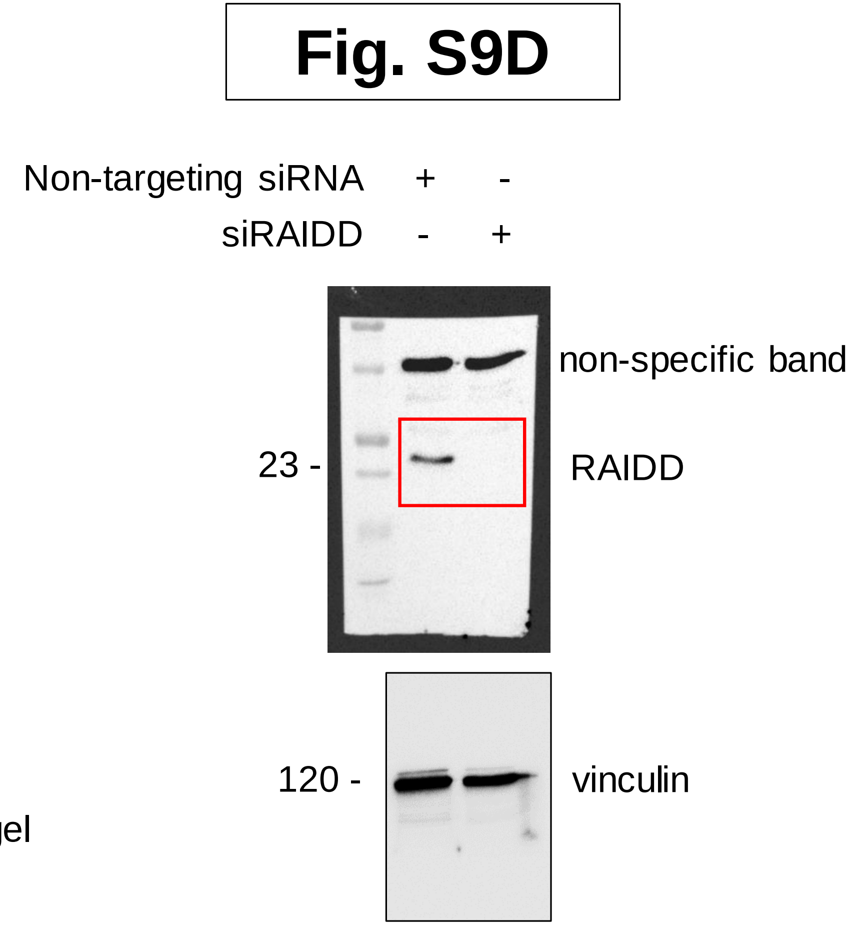
Fig. S9D
Non-targeting siRNA + -
siRAIDD - +
non-specific band
23 -
RAIDD
120 -
vinculin

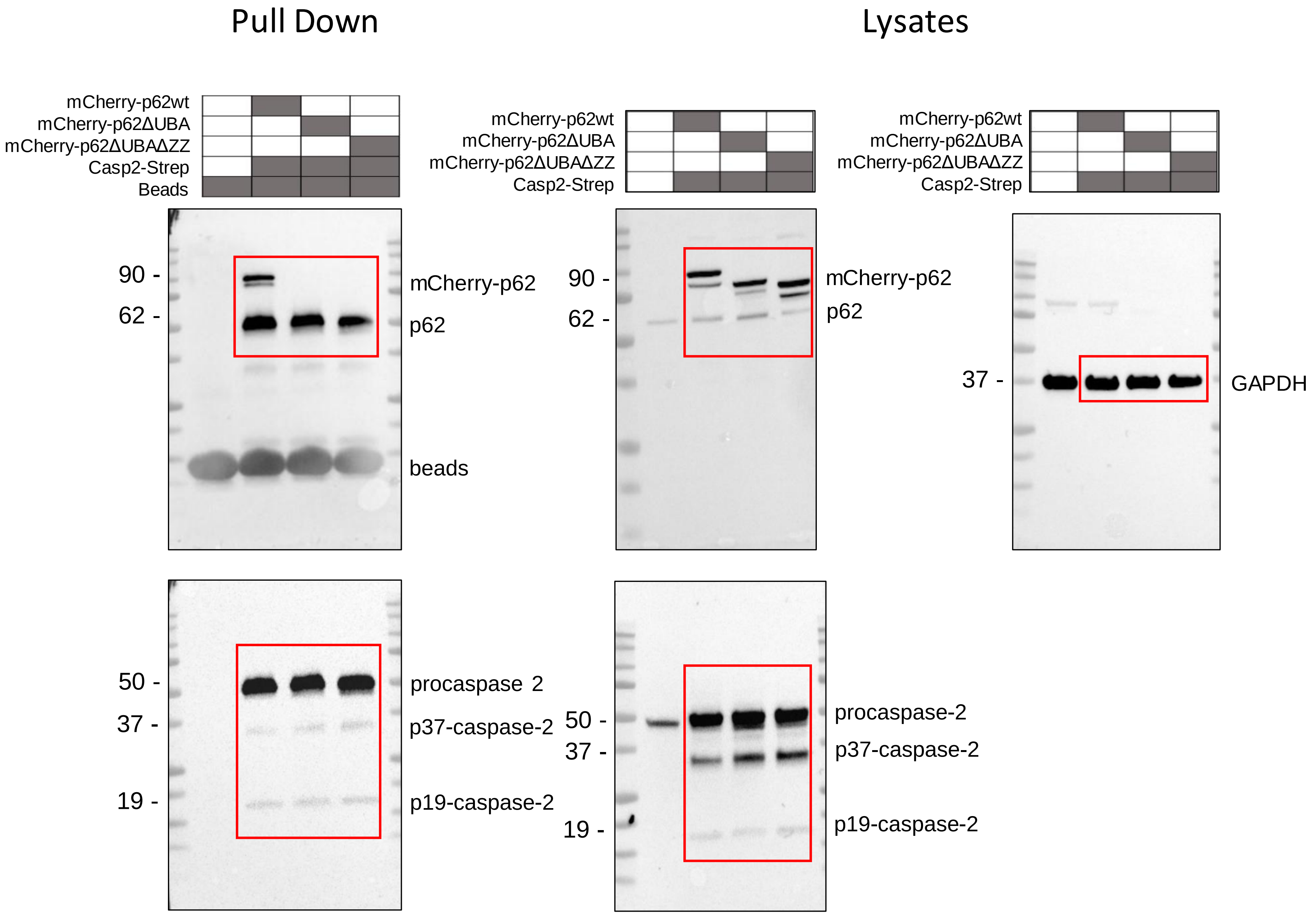
Pull Down
Lysates
mCherry-p62wt
mCherry-p62ΔUBA
mCherry-p62ΔUBAΔZZ
Casp2-Strep
Beads
90 -
62 -
mCherry-p62
p62
beads
50 -
37 -
19 -
procaspase 2
p37-caspase-2
p19-caspase-2
37 -
GAPDH
procaspase-2

Fig. 7D

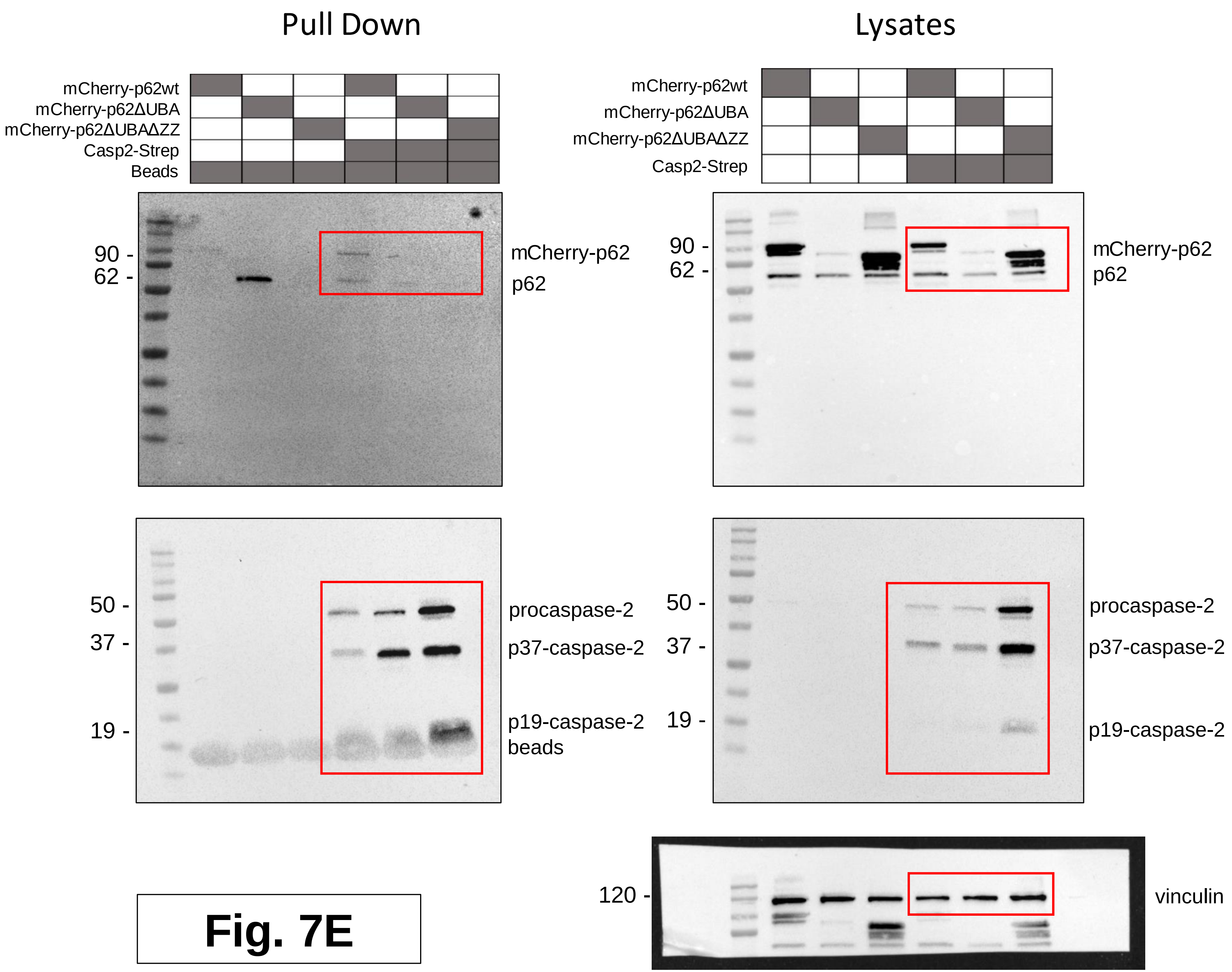

Pull Down
Lysates
mCherry-p62wt
mCherry-p62ΔUBA
mCherry-p62ΔUBAΔZZ
Casp2-Strep
Beads
90 -
62 -
mCherry-p62
p62
50 -
37 -
19 -
procaspase-2
p37-caspase-2
p19-caspase-2
beads
120 -
vinculin

Fig. 7E
